# Exploring *q*-Bernstein-Bézier surfaces in Minkowski space: Analysis, modeling, and applications

**DOI:** 10.1371/journal.pone.0299892

**Published:** 2024-05-30

**Authors:** Sadia Bashir, Daud Ahmad, Ghada Ali

**Affiliations:** 1 Department of Mathematics, University of the Punjab, Lahore, Pakistan; 2 Department of Mathematics, King Abdulaziz University Jeddah, Saudi Arabia; University of the West of Scotland, UNITED KINGDOM

## Abstract

In this paper, we examine *q*-Bernstein-Bézier surfaces in Minkowski space-$R_{1}^{3}$ with *q* as the shape parameter. These surfaces, a generalization of Bézier surfaces, have applications in mathematics, computer-aided geometric design, and computer graphics for the surface formation and modeling. We analyze the timelike and spacelike cases of *q*-Bernstein-Bézier surfaces using known boundary control points. The mean curvature and Gaussian curvature of these *q*-Bernstein-Bézier surfaces are computed by finding the respective fundamental coefficients. We also investigate the shape operator dependency for timelike and spacelike *q*-Bernstein-Bézier surfaces in Minkowski space-$R_{1}^{3}$, and provide biquadratic and bicubic *q*-Bernstein-Bézier surfaces as illustrative examples for different values of the shape controlling parameter *q*.

## 1 Introduction

Mathematical models are used to describe a number of physical phenomena as well as the geometry of a structure. These models play an important role in understanding and designing a desired geometric structure: including architecture of 3-dimensional models of buildings, automotive and automobiles, aerospace technology to design an aircraft or a spacecraft, shipbuilding (geometry of floating vessels), geoscience for particular types of maps and the geometry of the molecular structures studied in Chemistry. The surfaces following certain constraint structure and the geometric properties of the surfaces find their applications in computer aided-manufacturing (CAM), computer aided-designs (CAD) and computer aided-geometric designs (CAGD). Curves and surfaces are the primary tools of (CAM/CAD/CAGD) systems, and they deliver information about the geometry and shape of the artifacts. For the construction of a curve or a surface, the appropriate form is its parametric representation. It has its dominance over the other representations of the curves or the surfaces when the prescribed boundary is given by the control points. The parametric representation is sufficiently flexible to control the shape of the curve and the surface and it is more convenient in its use to study the geometry of the surfaces rather than when a surface is expressed in non-parametric form. A regular surface is a geometric object which restricts sharp edges and self-intersections and it is mathematically represented by a function of two parameters, usually called the surface parameters. In computer graphics (CG) and (CAD) systems, the curves and surfaces are usually expressed as the parametric-polynomials along with control points. The polynomial curves and polynomial surfaces depend on the bases functions. The restricted class of such curves and surfaces are the Bézier-curves and Bézier-surfaces [1]. These curves and surfaces were initially used by Pierre-Bézier in designing the needed auto-surfaces. De-Casteljau algorithm is used for repeated linear interpolation of the control points for the desired Bézier curves and surfaces in Bernstein bases form. The Bernstein polynomials [2] as the weights of the Bézier curves and the surfaces control their shape for the prescribed network of the control-points. Bézier-surfaces have a set of algorithmic-properties which can be used to analyze and interpret the shapes. The Bézier-surfaces formed by the product of two Bézier-curves have the same properties as well. Bézier surfaces have found numerous applications [3, 4] across various disciplines, particularly in optimization theory, where they are utilized to find minimal Bézier surfaces, serving as extremals constrained by energy functionals in both Euclidean-$R^{3}$ and Minkowski space-$R_{1}^{3}$ [5–12]. These surfaces show promise as candidates for optimization studies. This variational instance can be observed when deriving the EFEs as the result of vanishing variation of the Einstein-Hilbert action [13–15]. These surfaces can be further analyzed for the vanishing mean curvature condition that leads to the PDEs, which can be utilized to uncover the symmetries of the surfaces. For instance, discussions related to Lie symmetries can be found in the works [16–20].

Based on Bernstein-polynomials, several generalized-versions of the Bézier-surfaces are in use. One of such generalization of the classical-Bernstein polynomial, *q*-Bernstein polynomial (*q*, an integer) is admitted by Phillips [21, 22] (for its basic properties) and Oruc and Phillips [22] for the parametric representation of the curves. Other representation of the *q*-Bernstein-polynomials is by Kim [23], given as a linear-combination of higher-order polynomials. Kim representation of the *q*-Bernstein-polynomials is referred to as *q*-extension of the Bernstein-polynomials and they differ from Phillips representation of *q*-Bernstein-polynomials. The Kim [23] version used for *q*-Bernstein-polynomials facilitates one to find the derivatives in terms of lower-degree polynomials. Simsek and Acikgoz [24] addressed a new approach for generating new functions which produces the *q*-Bernstein type-polynomials. This construction differs from many of the previous-constructions in that they all used a recursion-formula. Sometimes the constructed Bézier-curves and Bézier-surfaces need to have their shapes changed in order to suit the requirements of our model. Apart from their use in optimization theory, the shape operator properties of these surfaces are also important when we introduce basis-functions with shape-control parameters. Khan [25] introduced a new class of curves and surfaces recognized as (*p*, *q*)-Bernstein-Bézier curves and (*p*, *q*)-Bernstein-Bézier surfaces [26] and surfaces that are an extension of *q*-Bernstein-Bézier curves and *q*-Bernstein-Bézier surfaces respectively. With the help of the parameters *p* and *q*, curves and surfaces can be modified in shape without changing the position of control-points. They also discussed some of its properties like partition of unity, end point property and non-negativity. Ahmad et al. [27] have discussed a computational scheme as a model for a quasi-minimal surface for the *q*-Bernstein Bézier-surface in the $R^{3}$-Euclidian-space which can be extended for the Minkowski space or alternatively the equivalent Euler Lagrange equation, the partial differential equation can be solved using the technique [28–30].

On the other hand, H. Minkowski [31] made an initial contribution to address the geometry of the objects moving in four dimensional spacetime in relativity (special and general relativity), in which space coordinates and time coordinates are mixed together and are not separable in the Riemannian and pseudo-Riemannian metrics. The Minkowski space comprises three space coordinates (namely *x*, *y*, *z*) and the time coordinate (*ct*-coordinate) is taken as the fourth coordinate. However, there is resemblance between the Euclidean and the Minkowski space while defining the distance concept. This enables one to find the surfaces in 3-dimensional Minkowski space. The metric element for the three-dimensional Minkowski space [32] is $<,>=dx_{1}^{2}+dx_{2}^{2}-dx_{3}^{2}$, where (*x*_1_, *x*_2_, *x*_3_) are the canonical-coordinates in Minkowski space-$R_{1}^{3}$. The Lorentz-Minkowski metric in Minkowski space-$R_{1}^{3}$ separates the regions into three types of vectors, they are timelike-vectors, lightlike-vectors and spacelike-vectors. In the light-like region of the Minkowski space, the null-vectors, pseudo-null-curves, null-curves, marginally trapped surfaces, B-scrolls pose, measuring the angular displacement is obscure. Many others have studied and analyzed timelike and spacelike surfaces in Minkowski space-$R_{1}^{3}$ in different disciplines of interest in science. Treibergs [33] has investigated spacelike hypersurfaces of constant mean-curvature in Minkowski space-$R_{1}^{3}$. For timelike-surfaces with a defined Gauss-map, Aledo et al. [34] have examined Lelievvre-type representation. In Minkowski space-$R_{1}^{3}$, Abdel-Baky and Abd-Ellah [35] investigated both (spacelike or timelike) governed *W*-surfaces. Brander et al. [36] used the non-compact real form SU to construct spacelike constant-mean curvature surfaces in Minkowski space-$R_{1}^{3}$. Lin [37] studied the impacts of curvature restrictions on the timelike-surfaces in Minkowski space-$R_{1}^{3}$ that are convex in the same way as are the surfaces in the Euclidean space-$R^{3}$. Kossowski [38] obtained zero-mean curvature surface constraints in Minkowski space-$R_{1}^{3}$. Georgiev [39] found sufficient conditions for the spacelike Bézier surfaces. Kuşak Samancı and Celik [40] analyzed the geometric characteristics such as shape operator, Gauss curvature and mean curvature of the Bézier surfaces in Minkowski space. In addition, related studies by Ceylan [41] focus on the geometry of Bézier curves in Minkowski space. Kılıçoglu and Şenyurt [42] investigate methods for determining Bézier curves when their derivatives are given. Kılıçoğlu and Yurttançıkmaz [43] explore Bézier curve representation of exponential curves. These studies provide valuable insights into various aspects of differential geometry, including our investigation into *q*-Bernstein surfaces.

In this work, we investigate a specialized and important class of surfaces that are utilized in computer graphics, the *q*-Bernstein-Bézier surface in Minkowski space-$R_{1}^{3}$. The objective is to determine the fundamental coefficients for the Gaussian curvature, mean curvature, and shape operator of timelike and spacelike *q*-Bernstein-Bézier surfaces in the Minkowski space. The obtained results are then applied to the corresponding shape operator of the biquadratic and bicubic (timelike/spacelike) *q*-Bernstein-Bézier surfaces in the Minkowski space, using *q* as the shape controlling parameter, to demonstrate the scheme.

The paper is organized as follows: Section 2 gives some preliminaries related to our work to make this paper self contained. In section 3, we demonstrate the shape operators of the non-degenerate cases of *q*-Bernstein-Bézier surfaces (qbbs) in Minkowski space-$R_{1}^{3}$. For the illustration of the scheme work of section 3, we provide numeric work related to the construction of biquadratic and bicubic (timelike/spacelike) *q*-Bernstein-Bézier surfaces (qbbs) in the section 4. Section 5 comprises final remarks and a glimpse of future work.

## 2 Preliminaries

In this section, we review basic concepts that will be later used in the work. In the Minkowski space, $R_{1}^{3}=(R^{3},\varphi_{L}\left(,\right))$, the Lorentzian-inner product of the two vectors ***α*** and ***β*** with the metric signature (2,1), is defined as,

$$\begin{array}{c} \varphi_{L}\left(\alpha ,\beta \right)=\alpha^{1}\beta^{1}+\alpha^{2}\beta^{2}-\alpha^{3}\beta^{3}, \end{array}$$ (2.1)

where ***α*** = (*α*^1^, *α*^2^, *α*^3^) and ***β*** = (*β*^1^, *β*^2^, *β*^3^) are the vectors in 3-dimensional space. A 3-vector ***β*** in Minkowski space-$R_{1}^{3}$ is referred to as

$$\begin{array}{c} \beta =\{\begin{array}{cc} \text{a}\;\text{spacelike}\;\text{vector}\;\text{if}, & \varphi_{L}\left(\beta ,\beta \right)>0\text{,}\\ \text{a}\;\text{lightlike}\;\text{vector}\;\text{if}, & \varphi_{L}\left(\beta ,\beta \right)=0\text{,}\\ \text{a}\;\text{timelike}\;\text{vector}\;\text{if}, & \varphi_{L}\left(\beta ,\beta \right)<0\text{.} \end{array} \end{array}$$ (2.2)

The timelike and the spacelike vectors are the non-degenerate vectors in the Minkowski space-$R_{1}^{3}$. The cross-product of two vectors ***α*** and ***β*** in Minkowski space-$R_{1}^{3}$ is

$$\begin{array}{c} \alpha \wedge_{L}\beta =-|\begin{array}{ccc} e^{1} & e^{2} & -e^{3}\\ \alpha^{1} & \alpha^{2} & \;\;\alpha^{3}\\ \beta^{1} & \beta^{2} & \;\;\beta^{3} \end{array}|, \end{array}$$ (2.3)

where $\wedge_{L}$ denotes the Lorentzian cross product in the Minkowski space-$R_{1}^{3}$. Let *M* be a surface represented by a regular parameterized surface $s:U\subset R^{2}\to R_{1}^{3}$ defined by **s** = **s**(*u*, *v*) of class *c*^*m*^ for *m* ≥ 1, in the Minkowski space-$R_{1}^{3}$. Let $T_{P}\left(M\right)$ be the tangent plane at a point P on the surface *M* spanned by the tangent vectors to the coordinate curves **s**(*u*, *v*_0_) and **s**(*u*_0_, *v*). Then the unit normal **N**(*u*, *v*) at the point P(s(u,v)) on the surface *M* is the vector field, given by

$$\begin{array}{c} N(u,v)=\frac{s_{u}\;\wedge_{L}\;s_{v}}{\left\|s_{u}\;\wedge_{L}\;s_{v}\right\|}=\frac{s_{u}\;\wedge_{L}\;s_{v}}{\sqrt{-\eta (E\;G-F^{2})}}. \end{array}$$ (2.4)

The first fundamental form on the plane $T_{P}(M)$ at the point P of the surface *M* corresponds to the matrix,

$$\begin{array}{c} \omega =(\begin{array}{cc} E & F\\ F & G \end{array}),\;where\;\text{det}\;(\omega )=EG-F^{2}, \end{array}$$ (2.5)

and *E*, *F*, *G* are the coefficients of the first-fundamental form of the surface **s**(*u*, *v*) defined by

$$\begin{array}{c} E=\varphi_{L}(s_{u},s_{u}),\;F=\varphi_{L}(s_{u},s_{v}),\;G=\varphi_{L}(s_{v},s_{v}), \end{array}$$ (2.6)

for the non-degenerate surfaces (timelike or the spacelike surface) in Minkowski space-$R_{1}^{3}$. For a spcelike surface, det (*ω*) > 0 and for a timelike surface, det (*ω*) < 0. Non-degenerate surfaces (a timelike or a spacelike surface) in Minkowski space-$R_{1}^{3}$ are characterized by the term $\varphi_{L}(N,N)=\eta$. For a spacelike-surface, the normal **N** is a timelike vector as the tangent-plane $T_{P}(M)$ is spacelike, and thus, $\varphi_{L}(N,N)=\eta =-1$, whereas for a timelike-surface, the normal **N** is a spacelike-vector as the tangent-plane $T_{P}(M)$ is timelike, and thus in this case, $\varphi_{L}(N,N)=\eta =1$. So that, Lorentzian cross-product (Eq (2.3)) of vectors **s**_*u*_ and **s**_*v*_ yields,

$$\begin{array}{c} \|s_{u}\;\wedge_{L}\;s_{v}\|=\sqrt{-\eta (E\;G-F^{2})}=\sqrt{-\eta \;\text{det}\;\omega }. \end{array}$$ (2.7)

The coefficients *e*, *f*, *g* of second-fundamental form of the surface *M* on a tangent-plane $T_{P}(M)$ can be computed by using the Lorentzian-inner product (Eq (2.1)) of the unit normal vector **N** with that of the second order partial derivatives **s**_*uu*_, **s**_*uv*_, **s**_*vv*_ of the regular parameterized surface **s**,

$$\begin{array}{c} e=\varphi_{L}(s_{uu},N),\;f=\varphi_{L}(s_{uv},N),\;g=\varphi_{L}(s_{vv},N). \end{array}$$ (2.8)

Finding the fundamental coefficients *E*, *F* and *G*, *e*, *f*, and *g* (Eqs (2.6) and (2.8)) enables one to find the matrix *V* = (*b*_*jk*_)_2×2_, where

$$\begin{array}{c} V=(\begin{array}{cc} b_{11} & b_{12}\\ b_{21} & b_{22} \end{array})={\left(\begin{array}{cc} E & F\\ F & G \end{array}\right)}^{-1}(\begin{array}{cc} e & f\\ f & g \end{array})={\left(\text{det}\;\omega \right)}^{-1}(\begin{array}{cc} eG-fF & fG-gF\\ fE-eF & gE-fF \end{array}), \end{array}$$ (2.9)

corresponds to the shape operator of the surface **s**(*u*, *v*). From the above matrix (2.9) related to the shape operator of the surface, one can compute the mean-curvature *H* and the Gauss-curvature *K* of the surfaces in its non-degenerate case (a spacelike or a timelike surface) as follows,

$$\begin{array}{c} H=\frac{1}{2}\;\eta \;\text{tr}\left(V\right)=\frac{1}{2}\;\eta \;(\frac{eG-2fF+Eg}{EG-F^{2}}),\;K=\eta \;\text{det}\left(V\right)=\eta \;\frac{eg-F^{2}}{EG-F^{2}}. \end{array}$$ (2.10)

**Definition 2.1**. A Bézier curve in Euclidian space-$R^{3}$ over the (*m* + 1)-control points, ♭_0_, ♭_1_,…, ♭_*m*_ is given by

$$\begin{array}{c} B\left(v\right)=\sum_{ȷ=0}^{m}B_{ȷ}^{m}\left(v\right){♭}_{ȷ}, \end{array}$$ (2.11)

where $B_{ȷ}^{m}\left(v\right)$ is the *m*^*th*^ degree classical Bernstein-polynomial,

Bȷm(v)=Cȷmvȷ(1-v)m-ȷ,whereCȷm=(mȷ),for0≤m≤ȷ. (2.12)

**Definition 2.2**. A Bézier surface in Euclidian space-$R^{3}$, along with classical Bernstein bases functions, $B_{ȷ}^{m}\left(u\right)$ and $B_{\ell }^{n}\left(v\right)$ (Eq (2.12)) of *m*^*th*^ and *n*^*th*^ degree respectively, and control points ${♭}_{00}$, ${♭}_{01}$,…, ${♭}_{mn}$, can be written in the form for 0≤m≤ȷ and 0 ≤ *n* ≤ *ℓ*

$$\begin{array}{c} B\left(u,v\right)=\sum_{ȷ,\ell =0}^{m,n}B_{ȷ}^{m}\left(u\right)B_{\ell }^{n}\left(v\right){♭}_{ȷ\ell },\;\left(u,v\right)\in \left[0,1\right]\times \left[0,1\right]. \end{array}$$ (2.13)

**Definition 2.3**. A *q*-Bernstein-Bézier curve (qbbc) over the (*n* + 1)-control points ♭_0_, ♭_1_,…, ♭_*n*_, in Euclidian space-$R^{3}$, is given by

$$\begin{array}{c} s\left(v\right)=\sum_{ȷ=0}^{n}Q_{ȷ}^{n,q}\left(v\right){♭}_{ȷ},\;\text{for}\;v\in \left[0,1\right]\;\text{and}\;q\in \left[0,1\right], \end{array}$$ (2.14)

where $Q_{ȷ}^{n,q}\left(v\right)$ is the *n*^*th*^ degree *q*-Bernstein-polynomial, for the shape controlling parameter *q*,

$$\begin{array}{c} Q_{ȷ}^{n,q}(v)=C_{ȷ}^{n}{\left[v\right]}_{q}^{ȷ}{\left[1-v\right]}_{\frac{1}{q}}^{n-ȷ},\;\text{for}\;0\leq n\leq ȷ, \end{array}$$ (2.15)

where

**Fig 1:**
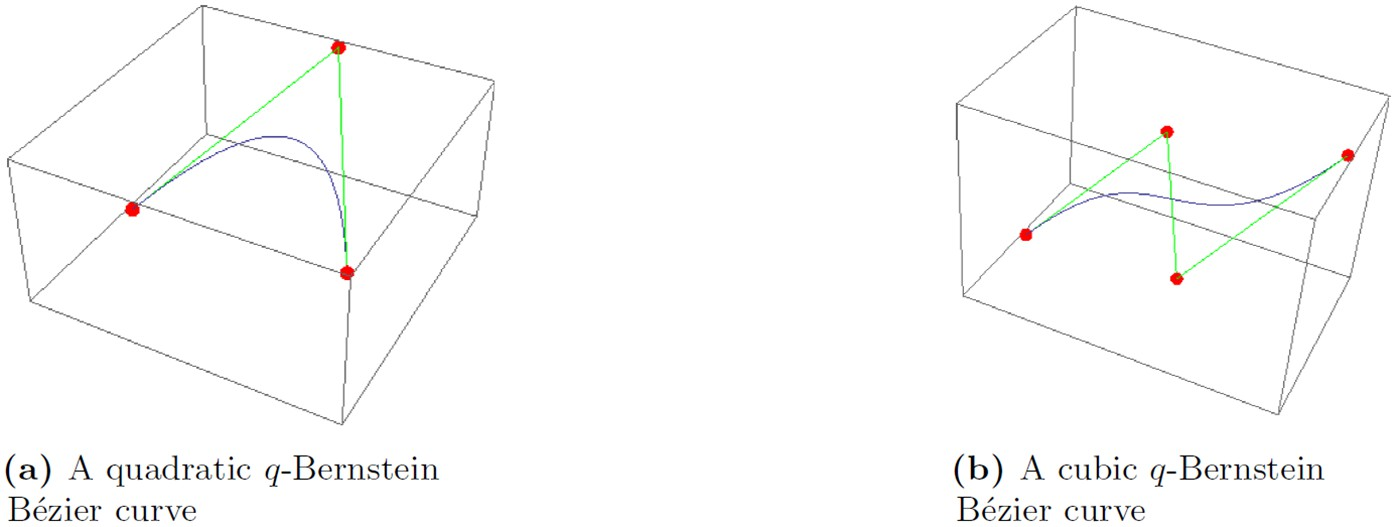
A quadratic and a cubic curve for *q* = 0.2 for the respective *q*-Bernstein Bézier curve for *n* = 2 and *n* = 3.

**Fig 2:**
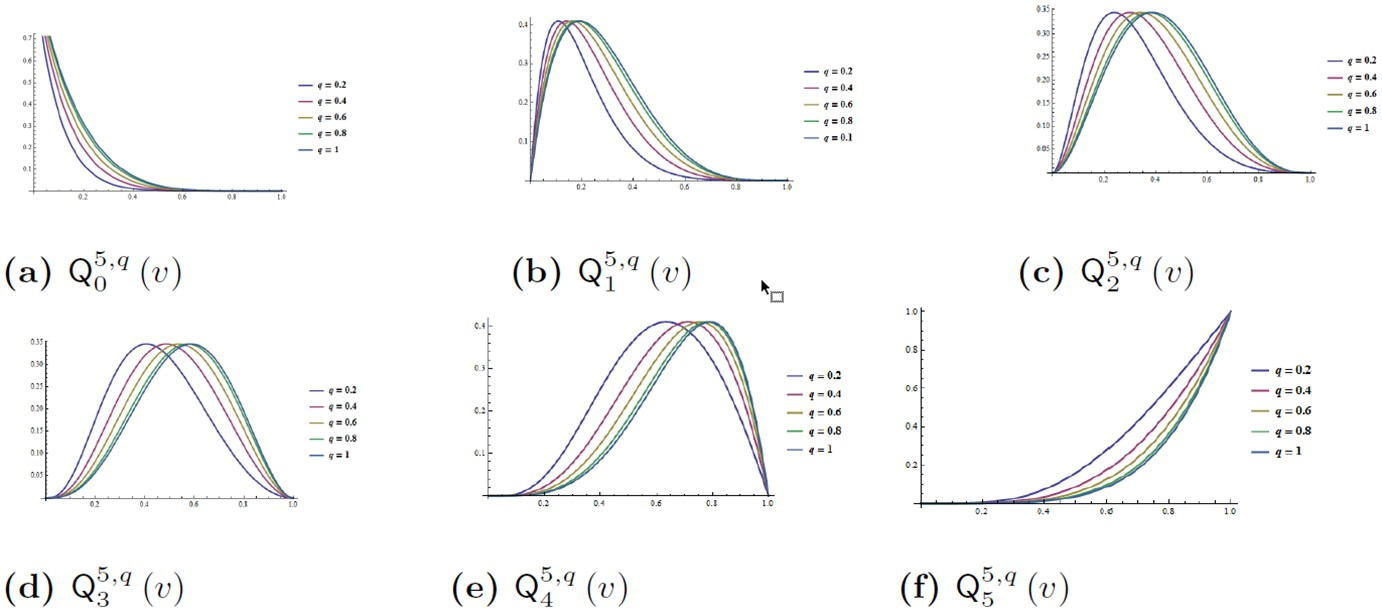
Fifth degree *q*-Bernstein polynomials for the shape controlling parameter *q*, for *q* = 0.2, 0.4, 0.6, 0.8, 1.

[v]qȷ=(1-qv)ȷ(1-q)ȷ,Cȷn=(nȷ), (2.16)

and consequently, ${\left[1-v\right]}_{\frac{1}{q}}^{n-ȷ}=\frac{{\left(1-{\left(\frac{1}{q}\right)}^{\left(1-v\right)}\right)}^{n-ȷ}}{{\left(1-(\frac{1}{q})\right)}^{n-ȷ}}$. In particular, commonly known quadratic and cubic *q*-Bernstein-Bézier curves (qbbc) determined from the Eq (2.14) (for *n* = 2 and *n* = 3 respectively) are shown in Fig 1. Note that, *q*-Bernstein-polynomials of degree *n* = 5 can be computed from Eq (2.15), shown in Fig 2 for *q*, for *q* = 0.2, 0.4, 0.6, 0.8, 1.

Let ♭_*ȷ*_ be the respective control-points of the curve obtained from Eq (2.14) for the *q*-Bernstein-Bézier curve in the Euclidian space-$R^{3}$. Then, its first order derivative *w*.*r*.*t*. the curve parameter *v* comes out as,

$$\begin{array}{c} s_{v}(v)=\sum_{ȷ=0}^{m}{\left(Q_{ȷ}^{m,q}\left(v\right)\right)}_{v}{♭}_{ȷ}=\sum_{ȷ=0}^{m-1}Q_{ȷ}^{m-1,q}(v){♭}_{ȷ}^{\left(1\right)}, \end{array}$$ (2.17)

where ${\left(Q_{ȷ}^{m,q}\left(v\right)\right)}_{v}$ represents the derivative of *q*-Bernstein-polynomial $Q_{ȷ}^{m,q}\left(v\right)$ with respect to *v* and it is defined by (for the detail see the ref. [27], Eqs (3.10) to (3.27))

$$\begin{array}{c} {\left(Q_{ȷ}^{m,q}\left(v\right)\right)}_{v}=m(Q_{j,q}^{m-1}(v)-Q_{j-1,q}^{m-1}(v))(\frac{q^{v}\;\text{log}\;q}{1-q}), \end{array}$$ (2.18)

and

$$\begin{array}{c} {♭}_{ȷ}^{\left(1\right)}=m(\frac{q^{v}\;\text{log}\;q}{1-q})({♭}_{ȷ+1}-{♭}_{ȷ}). \end{array}$$ (2.19)

It is to be remarked that the derivative of the *q*-Bernstein-polynomial appears as the polynomials of lesser degree (see Eq (2.18)) and the control points as the forward differences in the respective coordinates (see Eq (2.19)).

**Definition 2.4**. A *q*-Bernstein-Bézier surface **s**(*u*, *v*) is the tensor-product of *q*-Bernstein bases-functions $Q_{ȷ}^{m,q}\left(u\right)$ and $Q_{\ell }^{n,q}\left(v\right)$ (Eq (2.15)) along with the control-points ♭_00_, ♭_01_,…, ♭_*mn*_ in Euclidian space-$R^{3}$ and it is represented as

$$\begin{array}{c} s\left(u,v\right)=\sum_{ȷ,\ell =0}^{m,n}Q_{ȷ}^{m,q}\left(u\right)Q_{\ell }^{n,q}\left(v\right){♭}_{ȷ\ell },\;\text{for}\;0\leq m\leq ȷ,\;0\leq n\leq \ell ,\;\left(u,v\right)\in \left[0,1\right]\times \left[0,1\right]. \end{array}$$ (2.20)

The coordinate curves (usually called *u*-parameter or the *v*-parameter curves) on the *q*-Bernstein-Bézier surface **s**(*u*, *v*) can be determined by choosing one of the surface parameters as the constant. They are in the form **s**(*u*, *v*_0_) or **s**(*u*_0_, *v*). The coordinate curves **s**(*u*, 0), **s**(*u*, 1), **s**(0, *v*) and **s**(1, *v*) are the *q*-Bernstein-Bézier curves (compare it with Eq (2.14)). The coordinate curves **s**(*u*, 0), **s**(*u*, 1), **s**(0, *v*) and **s**(1, *v*) comprise the four edges of the *q*-Bernstein-Bézier surfaces (qbbs) along with the endpoint interpolation at the corner-points,

$$\begin{array}{c} s\left(0,0\right)={♭}_{00},\;s\left(1,0\right)={♭}_{m0},\;s\left(0,1\right)={♭}_{0m}\;s\left(1,1\right)={♭}_{mn}. \end{array}$$ (2.21)

It is to be noted that a *q*-Bernstein-Bézier surface (qbbs) is invariant under a three dimensional affine transformation L by virtue of the following equality

$$\begin{array}{c} L(\sum_{ȷ,\ell =0}^{m,n}Q_{ȷ}^{m,q}\left(u\right)Q_{\ell }^{n,q}\left(v\right){♭}_{ȷ\ell })=\sum_{ȷ,\ell =0}^{m,n}Q_{ȷ}^{m,q}\left(u\right)Q_{\ell }^{n,q}\left(v\right)L({♭}_{ȷ\ell }). \end{array}$$ (2.22)

Now, we present several results pertaining to the partial derivatives of the *q*-Bernstein-Bézier surface **s**(*u*, *v*). First order partial-derivative **s**_*u*_(*u*, *v*) of the *q*-Bernstein-Bézier surface **s**(*u*, *v*) (Eq (2.20)), with respect to the surface parameter *u* is

$$\begin{array}{c} s_{u}\left(u,v\right)=\sum_{ȷ,\ell =0}^{m-1,n}Q_{ȷ}^{m-1,q}(u)Q_{\ell }^{n,q}(v){♭}_{ȷ\ell }^{\left(1,0\right)}, \end{array}$$ (2.23)

where

$$\begin{array}{c} {♭}_{ȷ\ell }^{\left(1,0\right)}=m(\frac{q^{u}\;\text{log}\;q}{1-q})({♭}_{ȷ+1,\ell }-{♭}_{ȷ,\ell }). \end{array}$$ (2.24)

In the similar manner, the partial derivative of the first order of qbbs, *q*-Bernstein-Bézier surface, *w.r.t* the surface parameter *v* is

$$\begin{array}{c} s_{v}\left(u,v\right)=\sum_{ȷ,\ell =0}^{m,n-1}Q_{ȷ}^{m,q}(u)Q_{\ell }^{n-1,q}(v){♭}_{ȷ\ell }^{\left(0,1\right)}, \end{array}$$ (2.25)

where

$$\begin{array}{c} {♭}_{ȷ\ell }^{\left(0,1\right)}=n(\frac{q^{v}\;\text{log}\;q}{1-q})({♭}_{ȷ,\ell +1}-{♭}_{ȷ,\ell }). \end{array}$$ (2.26)

One can compute the partial derivatives of first order of the *q*-Bernstein-Bézier surface **s**(*u*, *v*) with surface parameter *u* and *v*, at the minimum-point (*u*, *v*) = (0, 0), from the Eqs (2.23)–(2.26),

$$\begin{array}{c} s_{u}\left(0,0\right)=m(\frac{q^{u}\;\text{log}\;q}{1-q})({♭}_{10}-{♭}_{00})={♭}_{00}^{\left(1,0\right)}, \end{array}$$ (2.27)

and

$$\begin{array}{c} s_{v}\left(0,0\right)=n(\frac{q^{v}\;\text{log}\;q}{1-q})({♭}_{01}-{♭}_{00})={♭}_{00}^{\left(0,1\right)}. \end{array}$$ (2.28)

From Eq (2.23), partial derivative of second order of *q*-Bernstein-Bézier surface **s**(*u*, *v*) *w*.*rt*. *u* is

$$\begin{array}{c} s_{uu}\left(u,v\right)=\sum_{ȷ,\ell =0}^{m-2,n}Q_{ȷ}^{m-2,q}(u)Q_{\ell }^{n,q}(v){♭}_{ȷ\ell }^{\left(2,0\right)}, \end{array}$$ (2.29)

where

$$\begin{array}{c} {♭}_{ȷ\ell }^{\left(2,0\right)}=m(m-1){\left(\frac{q^{u}\;\text{log}\;q}{1-q}\right)}^{2}({♭}_{ȷ+2,\ell }-2{♭}_{ȷ+1,\ell }+{♭}_{ȷ,\ell }). \end{array}$$ (2.30)

Similarly, using Eq (2.23), mixed partial derivative of second order, of *q*-Bernstein-Bézier surface **s**(*u*, *v*) is

$$\begin{array}{c} s_{uv}\left(u,v\right)=\sum_{ȷ,\ell =0}^{m-1,n-1}Q_{ȷ}^{m-1,q}(u)Q_{\ell }^{n-1,q}(v){♭}_{ȷ\ell }^{\left(1,1\right)}, \end{array}$$ (2.31)

where,

$$\begin{array}{c} {♭}_{ȷ\ell }^{\left(1,1\right)}=m\;n\;q^{u+v}{\left(\frac{\text{log}\;q}{1-q}\right)}^{2}({♭}_{ȷ+1,\ell +1}-{♭}_{ȷ,\ell +1}-{♭}_{ȷ+1,\ell }+{♭}_{ȷ,\ell }). \end{array}$$ (2.32)

Now, from the Eq (2.25), partial derivative of second order, of *q*-Bernstein-Bézier surface (qbbs) *w*.*r*.*t*. parameter *v* is

$$\begin{array}{c} s_{vv}\left(u,v\right)=\sum_{ȷ,\ell =0}^{m,n-2}Q_{ȷ}^{m,q}(u)Q_{\ell }^{n-2,q}(v){♭}_{ȷ\ell }^{\left(0,2\right)}, \end{array}$$ (2.33)

where

$$\begin{array}{c} {♭}_{ȷ\ell }^{\left(0,2\right)}=n(n-1){\left(\frac{q^{v}\;\text{log}\;q}{1-q}\right)}^{2}({♭}_{ȷ,\ell +2}-2{♭}_{ȷ,\ell +1}+{♭}_{ȷ,\ell }). \end{array}$$ (2.34)

We can find now **s**_*uu*_(0, 0), **s**_*uv*_(0, 0) and **s**_*vv*_(0, 0), *second*-order partial derivatives of the *q*-Bernstein-Bézier surface **s**(*u*, *v*) with respect to the surface parameters *u* and *v* at the minimum point (*u*, *v*) = (0, 0). From Eqs (2.29) and (2.30), we find that

$$\begin{array}{c} s_{uu}\left(0,0\right)=m\left(m-1\right){\left(\frac{q^{u}\;\text{log}\;q}{1-q}\right)}^{2}({♭}_{20}-{♭}_{10}+{♭}_{00})={♭}_{00}^{\left(2,0\right)}, \end{array}$$ (2.35)

whereas **s**_*uv*_(0, 0) can be obtained from the Eqs (2.31) and (2.32),

$$\begin{array}{c} s_{uv}\left(0,0\right)=m\;n\;q^{u+v}{\left(\frac{\text{log}\;q}{1-q}\right)}^{2}({♭}_{11}-{♭}_{10}-{♭}_{01}+{♭}_{00})={♭}_{00}^{\left(1,1\right)}, \end{array}$$ (2.36)

and **s**_*vv*_(0, 0), from the Eqs (2.33) and (2.34) is

$$\begin{array}{c} s_{vv}\left(0,0\right)=n\left(n-1\right){\left(\frac{q^{v}\;\text{log}\;q}{1-q}\right)}^{2}({♭}_{02}-2{♭}_{01}+{♭}_{00})={♭}_{00}^{\left(0,2\right)}. \end{array}$$ (2.37)

The aforementioned derivatives are computed for the upcoming section 3 with the aim of determining the shape operator in non-degenerate cases (timelike/spacelike) of *q*-Bernstein-Bézier surfaces (qbbs) in Minkowski space. These derivatives can then be used in the framework presented in section 4, which focuses on numerical computations for the biquadratic and bicubic (timelike/spacelike) *q*-Bernstein-Bézier surfaces (qbbs).

## 3 Results and discussion

In this section, we find the metric coefficients of *q*-Bernstein-Bézier surface (qbbs) for the two cases, timelike and spacelike surfaces, by generalizing *q*-Bernstein-Bézier surface (qbbs) in Minkowski space-$R_{1}^{3}$. This enables us to find the Gauss-curvature and mean-curvature of the *q*-Bernstein-Bézier surface (qbbs) and the corresponding matrix form of the shape operator in Minkowski space-$R_{1}^{3}$.

**Definition 3.1**. A *q*-Bernstein-Bézier surface (qbbs), **s**(*u*, *v*) (where $s\left(u,v\right)=\sum_{ȷ,\ell =0}^{m,n}Q_{ȷ}^{m,q}\left(u\right)Q_{\ell }^{n,q}\left(v\right){♭}_{ȷ\ell }$) over the control-points ${\left\{{♭}_{ȷ\ell }\right\}}_{ȷ,\ell =0}^{m,n}$, as the tensor product of *q*-Bernstein-bases functions $Q_{ȷ}^{m,q}\left(u\right)$ and $Q_{\ell }^{n,q}\left(v\right)$ is indicated in the Eq (2.20). We are interested in the non-degenerate cases of the *q*-Bernstein-Bézier surfaces (qbbs). In the Minkowski space-$R_{1}^{3}$, *q*-Bernstein-Bézier surface, **s**(*u*, *v*) given by Eq (2.20) is said to be timelike if $\varphi_{L}(N,N)=1$ and spacelike if $\varphi_{L}(N,N)=-1$, where **N** is the unit normal to the surface.

**Theorem 3.1**. *It can readily be seen that coefficients E*, *F and G of the first fundamental form of the (timelike/spacelike) q-Bernstein-Bézier surface* **s**(*u*, *v*) *in Minkowski space*-$R_{1}^{3}$ *can be computed from the* Eq (2.6). *Thus the coefficient E* (*from the* Eq (2.6)) *of the first-fundamental form of the q-Bernstein-Bézier surface* (qbbs), *along with the first-order partial derivative* **s**_*u*_(*u*, *v*) *of the q-Bernstein-Bézier surface* (qbbs) *from the* Eq (2.23) *and the Lorentzian-inner product defined in* Eq (2.1), *is given by*

$$\begin{array}{c} \begin{array}{cc} E & =\varphi_{L}(s_{u}\left(u,v\right),s_{u}\left(u,v\right))\\ & ={\left(\sum_{ȷ,\ell =0}^{m-1,n}Q_{ȷ}^{m-1,q}(u)Q_{\ell }^{n,q}(v)x_{ȷ\ell }^{\left(1,0\right)}\right)}^{2}+{\left(\sum_{ȷ,\ell =0}^{m-1,n}Q_{ȷ}^{m-1,q}(u)Q_{\ell }^{n,q}(v)y_{ȷ\ell }^{\left(1,0\right)}\right)}^{2}-\\ & \;{\left(\sum_{ȷ,\ell =0}^{m-1,n}Q_{ȷ}^{m-1,q}(u)Q_{\ell }^{n,q}(v)z_{ȷ\ell }^{\left(1,0\right)}\right)}^{2}\\ & =\varphi_{L}(\sum_{ȷ,\ell =0}^{m-1,n}Q_{ȷ}^{m-1,q}(u)Q_{\ell }^{n,q}(v){♭}_{ȷ\ell }^{\left(1,0\right)},\sum_{ȷ,\ell =0}^{m-1,n}Q_{ȷ}^{m-1,q}(u)Q_{\ell }^{n,q}(v){♭}_{ȷ\ell }^{\left(1,0\right)}). \end{array} \end{array}$$ (3.1)

*In the similar way other coefficients F and G can be computed and they are*,

$$\begin{array}{c} F=\varphi_{L}(\sum_{ȷ,\ell =0}^{m-1,n}Q_{ȷ}^{m-1,q}(u)Q_{\ell }^{n,q}(v){♭}_{ȷ\ell }^{\left(1,0\right)},\sum_{ȷ,\ell =0}^{m,n-1}Q_{ȷ}^{m,q}(u)Q_{\ell }^{n-1,q}(v){♭}_{ȷ\ell }^{\left(0,1\right)}), \end{array}$$ (3.2)

$$\begin{array}{c} G=\varphi_{L}(\sum_{ȷ,\ell =0}^{m,n-1}Q_{ȷ}^{m,q}(u)Q_{\ell }^{n-1,q}(v){♭}_{ȷ\ell }^{\left(0,1\right)},\sum_{ȷ,\ell =0}^{m,n-1}Q_{ȷ}^{m,q}(u)Q_{\ell }^{n-1,q}(v){♭}_{ȷ\ell }^{\left(0,1\right)}). \end{array}$$ (3.3)

**Corollary 3.1.1**. *In the Minkowski space*-$R_{1}^{3}$, *the coefficients E*, *F*, *G* *of the first-fundamental form of the (timelike/spacelike) q-Bernstein-Bézier surface* (qbbs) *can be obtained from the* Eqs (3.1)–(3.3), *at the minimum point* (*u*, *v*) = (0, 0)

$$\begin{array}{c} E=\varphi_{L}({♭}_{00}^{\left(1,0\right)},{♭}_{00}^{\left(1,0\right)}),\;F=\varphi_{L}({♭}_{00}^{\left(1,0\right)},{♭}_{00}^{\left(0,1\right)}),\;G=\varphi_{L}({♭}_{00}^{\left(0,1\right)},{♭}_{00}^{\left(0,1\right)}). \end{array}$$ (3.4)

**Corollary 3.1.2**. *The first-fundamental form of the (timelike/spacelike) q-Bernstein-Bézier surface* (qbbs) *at the point* (*u*, *v*) = (0, 0) *in Minkowski space*-$R_{1}^{3}$ *is*

$$\begin{array}{c} \begin{array}{cc} ds^{2} & =\varphi_{L}(\sum_{ȷ,\ell =0}^{m-1,n}Q_{ȷ}^{m-1,q}(u)Q_{\ell }^{n,q}(v){♭}_{ȷ\ell }^{\left(1,0\right)},\sum_{ȷ,\ell =0}^{m-1,n}Q_{ȷ}^{m-1,q}(u)Q_{\ell }^{n,q}(v){♭}_{ȷ\ell }^{\left(1,0\right)})du^{2}\\ & +2\varphi_{L}(\sum_{ȷ,\ell =0}^{m-1,n}Q_{ȷ}^{m-1,q}(u)Q_{\ell }^{n,q}(v){♭}_{ȷ\ell }^{\left(1,0\right)},\sum_{ȷ,\ell =0}^{m,n-1}Q_{ȷ}^{m,q}(u)Q_{\ell }^{n-1,q}(v){♭}_{ȷ\ell }^{\left(0,1\right)})\;dudv\\ & +\varphi_{L}(\sum_{ȷ,\ell =0}^{m,n-1}Q_{ȷ}^{m,q}(u)Q_{\ell }^{n-1,q}(v){♭}_{ȷ\ell }^{\left(0,1\right)},\sum_{ȷ,\ell =0}^{m,n-1}Q_{ȷ}^{m,q}(u)Q_{\ell }^{n-1,q}(v){♭}_{ȷ\ell }^{\left(0,1\right)})dv^{2}. \end{array} \end{array}$$ (3.5)

**Corollary 3.1.3**. *The first-fundamental form of the (timelike/spacelike) q-Bernstein-Bézier surface* **s**(*u*, *v*) *at the minimum-point* (*u*, *v*) = (0, 0) *is obtained from the* Eq (3.5) *in Minkowski space*-$R_{1}^{3}$

$$\begin{array}{c} ds^{2}=\varphi_{L}({♭}_{00}^{\left(1,0\right)},{♭}_{00}^{\left(1,0\right)})\;du^{2}+\varphi_{L}({♭}_{00}^{\left(1,0\right)},{♭}_{00}^{\left(0,1\right)})\;dudv+\varphi_{L}({♭}_{00}^{\left(0,1\right)},{♭}_{00}^{\left(0,1\right)})\;dv^{2}. \end{array}$$ (3.6)

The components (*μ*_1_, *μ*_2_, *μ*_3_) of the numerator $\mu =s_{u}\;\wedge_{L}\;s_{v}$ of the unit normal **N** to the *q*-Bernstein-Bézier surface (qbbs) are,

$$\begin{array}{c} \begin{array}{cc} \mu_{1}= & \sum_{ȷ,\ell =0}^{m,n-1}Q_{ȷ}^{m,q}(u)Q_{\ell }^{n-1,q}(v)y_{ȷ\ell }^{\left(0,1\right)}\;\sum_{ȷ,\ell =0}^{m-1,n}Q_{ȷ}^{m-1,q}(u)Q_{\ell }^{n,q}(v)z_{ȷ\ell }^{\left(1,0\right)}-\\ & \sum_{ȷ,\ell =0}^{m-1,n}Q_{ȷ}^{m-1,q}(u)Q_{\ell }^{n,q}(v)y_{ȷ\ell }^{\left(1,0\right)}\;\sum_{ȷ,\ell =0}^{m,n-1}Q_{ȷ}^{m,q}(u)Q_{\ell }^{n-1,q}(v)z_{ȷ\ell }^{\left(0,1\right)}, \end{array} \end{array}$$ (3.7)

$$\begin{array}{c} \begin{array}{cc} \mu_{2}= & \sum_{ȷ,\ell =0}^{m-1,n}Q_{ȷ}^{m-1,q}(u)Q_{\ell }^{n,q}(v)x_{ȷ\ell }^{\left(1,0\right)}\;\sum_{ȷ,\ell =0}^{m,n-1}Q_{ȷ}^{m,q}(u)Q_{\ell }^{n-1,q}(v)z_{ȷ\ell }^{\left(0,1\right)}-\\ & \sum_{ȷ,\ell =0}^{m,n-1}Q_{ȷ}^{m,q}(u)Q_{\ell }^{n-1,q}(v)x_{ȷ\ell }^{\left(0,1\right)}\;\sum_{ȷ,\ell =0}^{m-1,n}Q_{ȷ}^{m-1,q}(u)Q_{\ell }^{n,q}(v)z_{ȷ\ell }^{\left(1,0\right)}, \end{array} \end{array}$$ (3.8)

$$\begin{array}{c} \begin{array}{cc} \mu_{3}= & \sum_{ȷ,\ell =0}^{m-1,n}Q_{ȷ}^{m-1,q}(u)Q_{\ell }^{n,q}(v)x_{ȷ\ell }^{\left(1,0\right)}\;\sum_{ȷ,\ell =0}^{m,n-1}Q_{ȷ}^{m,q}(u)Q_{\ell }^{n-1,q}(v)y_{ȷ\ell }^{\left(0,1\right)}-\\ & \sum_{ȷ,\ell =0}^{m,n-1}Q_{ȷ}^{m,q}(u)Q_{\ell }^{n-1,q}(v)x_{ȷ\ell }^{\left(0,1\right)}\;\sum_{ȷ,\ell =0}^{m-1,n}Q_{ȷ}^{m-1,q}(u)Q_{\ell }^{n,q}(v)y_{ȷ\ell }^{\left(1,0\right)}. \end{array} \end{array}$$ (3.9)

It is to be noted that in the results below, *η* = 1 for the timelike *q*-Bernstein-Bézier surface **s**(*u*, *v*) and *η* = −1 for the spacelike *q*-Bernstein-Bézier surface **s**(*u*, *v*).

**Theorem 3.2**. *In the Minkowski space*-$R_{1}^{3}$, *the normal vector-field* **N** *to the non-degenerate (timelike/spacelike) q-Bernstein-Bézier surface* **s**(*u*, *v*) *is given by*,

$$\begin{array}{c} N=\frac{\mu }{\sqrt{-\eta \;\varphi_{L}\left(\mu ,\mu \right)}}, \end{array}$$ (3.10)

*where* $\mu =s_{u}\;\wedge_{L}\;s_{v}$. *The components* (*μ*_1_, *μ*_2_, *μ*_3_) *of* ***μ*** *are given in* Eqs (3.7)–(3.9), *and η* = 1, *for the timelike q-Bernstein-Bézier surface* (qbbs) *and η* = −1 *if it is a spacelike-surface*.

*Proof*. In the Minkowski space-$R_{1}^{3}$, the unit normal **N** to the tangent plane $T_{P}\left(M\right)$ at the point P on the surface **s**(*u*, *v*) spanned by the tangent vectors **s**_*u*_ and **s**_*v*_ to the coordinate curves on the (timelike/spacelike) *q*-Bernstein-Bézier surfaces **s**(*u*, *v*), (by Lorentzian-cross product (as defined by the Eq (2.3) of the tangent vectors **s**_*u*_ and **s**_*v*_), is

$$\begin{array}{c} N=\frac{s_{u}\;\wedge_{L}\;s_{v}}{\left\|s_{u}\;\wedge_{L}\;s_{v}\right\|}. \end{array}$$ (3.11)

Plugging the values of the **s**_*u*_ and **s**_*v*_ from the Eqs (2.23) and (2.25) in the Eq (3.11), we get

$$\begin{array}{c} N=\frac{\sum_{ȷ=0}^{m-1}\sum_{\ell =0}^{n}Q_{ȷ}^{m-1,q}(u)Q_{\ell }^{n,q}(v){♭}_{ȷ\ell }^{\left(1,0\right)}\;\wedge_{L}\;\sum_{ȷ=0}^{m}\sum_{\ell =0}^{n-1}Q_{ȷ}^{m,q}(u)Q_{\ell }^{n-1,q}(v){♭}_{ȷ\ell }^{\left(0,1\right)}}{\left\|\sum_{ȷ=0}^{m-1}\sum_{\ell =0}^{n}Q_{ȷ}^{m-1,q}(u)Q_{\ell }^{n,q}(v){♭}_{ȷ\ell }^{\left(1,0\right)}\;\wedge_{L}\;\sum_{ȷ=0}^{m}\sum_{\ell =0}^{n-1}Q_{ȷ}^{m,q}(u)Q_{\ell }^{n-1,q}(v){♭}_{ȷ\ell }^{\left(0,1\right)}\right\|}, \end{array}$$ (3.12)

where $\varphi_{L}(N,N)=\eta$ for both the timelike and spacelike surfaces. For timelike *q*-Bernstein-Bézier surface **s**(*u*, *v*), $\varphi_{L}(N,N)=1$ whereas for the spacelike, $\varphi_{L}(N,N)=-1$ and the norm

$$\begin{array}{c} \|s_{u}\;\wedge_{L}\;s_{v}\|=\sqrt{\left|E\;G-F^{2}\right|}=\sqrt{-\eta (E\;G-F^{2})}=\sqrt{-\eta \;\text{det}\;(\omega )}. \end{array}$$ (3.13)

Thus, the surface-normal Eq (3.12), by virtue of the Eqs (2.1) and (3.13) can be written as

$$\begin{array}{c} N=\frac{\left(\mu_{1},\mu_{2},\mu_{3}\right)}{\sqrt{\left|\mu_{1}^{2}+\mu_{2}^{2}-\mu_{3}^{2}\right|}}=\frac{\left(\mu_{1},\mu_{2},\mu_{3}\right)}{\sqrt{-\eta (\mu_{1}^{2}+\mu_{2}^{2}-\mu_{3}^{2})}}=\frac{\mu }{\sqrt{-\eta \;\varphi_{L}(\mu ,\mu )}}. \end{array}$$ (3.14)

**Corollary 3.2.1**. *It can readily be seen that the normal-vector field* **N** *defined in* Eq (3.11) *of the (timelike/spacelike) q-Bernstein-Bézier surface* **s**(*u*, *v*) *in Minkowski space*-$R_{1}^{3}$ *at the min-point* (*u*, *v*) = (0, 0) *is*

$$\begin{array}{c} N\left(u,v\right)=\frac{{♭}_{00}^{\left(1,0\right)}\;\wedge_{L}\;{♭}_{00}^{\left(0,1\right)}}{\left\|{♭}_{00}^{\left(1,0\right)}\;\wedge_{L}\;{♭}_{00}^{\left(0,1\right)}\right\|}. \end{array}$$ (3.15)

**Theorem 3.3**. *The determinant of the first fundamental-form of the (timelike/spacelike) q-Bernstein-Bézier surface* **s**(*u*, *v*) *in Minkowski space*-$R_{1}^{3}$ *is*

$$\begin{array}{c} \text{det}\;\left(\omega \right)=-\varphi_{L}(\mu ,\mu ). \end{array}$$ (3.16)

*Proof*. Note that the determinant det (*ω*) = *EG* − *F*^2^ of the first-fundamental form of the (timelike/spacelike) *q*-Bernstein-Bézier surface (qbbs) in Minkowski space-$R_{1}^{3}$ is given by the Eq (2.5). Substituting the fundamental coefficients *E*, *F*, *G* (given by Eqs (3.1)–(3.3)) in this Eq (2.5), we find that

$$\begin{array}{c} \text{det}\left(\omega \right)=\left(\begin{array}{cc} & {\left(\begin{array}{cc} & \sum_{ȷ,\ell =0}^{m-1,n}Q_{ȷ}^{m-1,q}\left(u\right)Q_{\ell }^{n,q}\left(v\right)x_{ȷ\ell }^{\left(1,0\right)}\;\sum_{ȷ,\ell =0}^{m,n-1}Q_{ȷ}^{m,q}\left(u\right)Q_{\ell }^{n-1,q}\left(v\right)y_{ȷ\ell }^{\left(0,1\right)}-\\ & \sum_{ȷ,\ell =0}^{m,n-1}Q_{ȷ}^{m,q}\left(u\right)Q_{\ell }^{n-1,q}\left(v\right)x_{ȷ\ell }^{\left(0,1\right)}\;\sum_{ȷ,\ell =0}^{m-1,n}Q_{ȷ}^{m-1,q}\left(u\right)Q_{\ell }^{n,q}\left(v\right)y_{ȷ\ell }^{\left(1,0\right)} \end{array}\right)}^{2}-\\ & {\left(\begin{array}{cc} & \sum_{ȷ,\ell =0}^{m-1,n}Q_{ȷ}^{m-1,q}\left(u\right)Q_{\ell }^{n,q}\left(v\right)x_{ȷ\ell }^{\left(1,0\right)}\;\sum_{ȷ,\ell =0}^{m,n-1}Q_{ȷ}^{m,q}\left(u\right)Q_{\ell }^{n-1,q}\left(v\right)z_{ȷ\ell }^{\left(0,1\right)}-\\ & \sum_{ȷ,\ell =0}^{m,n-1}Q_{ȷ}^{m,q}\left(u\right)Q_{\ell }^{n-1,q}\left(v\right)x_{ȷ\ell }^{\left(0,1\right)}\;\sum_{ȷ,\ell =0}^{m-1,n}Q_{ȷ}^{m-1,q}\left(u\right)Q_{\ell }^{n,q}\left(v\right)z_{ȷ\ell }^{\left(1,0\right)} \end{array}\right)}^{2}-\\ & {\left(\begin{array}{cc} & \sum_{ȷ,\ell =0}^{m-1,n}Q_{ȷ}^{m-1,q}\left(u\right)Q_{\ell }^{n,q}\left(v\right)y_{ȷ\ell }^{\left(1,0\right)}\;\sum_{ȷ,\ell =0}^{m,n-1}Q_{ȷ}^{m,q}\left(u\right)Q_{\ell }^{n-1,q}\left(v\right)z_{ȷ\ell }^{\left(0,1\right)}-\\ & \sum_{ȷ,\ell =0}^{m,n-1}Q_{ȷ}^{m,q}\left(u\right)Q_{\ell }^{n-1,q}\left(v\right)y_{ȷ\ell }^{\left(0,1\right)}\;\sum_{ȷ,\ell =0}^{m-1,n}Q_{ȷ}^{m-1,q}\left(u\right)Q_{\ell }^{n,q}\left(v\right)z_{ȷ\ell }^{\left(1,0\right)} \end{array}\right)}^{2} \end{array}\right). \end{array}$$ (3.17)

The terms in above Eq (3.17) when compared with that of the components (*μ*_1_, *μ*_2_, *μ*_3_) (Eqs (3.7) to (3.9)) of the vector ***μ*** reduces it to the result stated in Eq (3.16).

**Corollary 3.3.1**. *The determinant* det (*ω*) *of the corresponding matrix* *ω* *of the first-fundamental form can be obtained directly from the fundamental coefficients* (3.4)

$$\begin{array}{c} \text{det}\left(\omega \right)=E\;G-F^{2}=\varphi_{L}({♭}_{00}^{\left(1,0\right)},{♭}_{00}^{\left(1,0\right)})\;\varphi_{L}({♭}_{00}^{\left(0,1\right)},{♭}_{00}^{\left(0,1\right)})-\varphi_{L}^{2}({♭}_{00}^{\left(1,0\right)},{♭}_{00}^{\left(0,1\right)}). \end{array}$$ (3.18)

*Note that the components* (*μ*_1_, *μ*_2_, *μ*_3_) (*Eqs* (3.7) *to* (3.9)) *of the vector* ***μ*** *for the (timelike/spacelike) q-Bernstein-Bézier surface* (qbbs) *at the point* (*u*, *v*) = (0, 0) *are*,

$$\begin{array}{c} \begin{array}{cc} & \mu_{1}=(y_{00}^{\left(0,1\right)}\;z_{00}^{\left(1,0\right)}-y_{00}^{\left(1,0\right)}\;z_{00}^{\left(0,1\right)}),\;\\ & \mu_{2}=-(x_{00}^{\left(0,1\right)}\;z_{00}^{\left(1,0\right)}-x_{00}^{\left(1,0\right)}\;z_{00}^{\left(0,1\right)}),\;\\ & \mu_{3}=-(x_{00}^{\left(0,1\right)}\;y_{00}^{\left(1,0\right)}-x_{00}^{\left(1,0\right)}\;y_{00}^{\left(0,1\right)}). \end{array} \end{array}$$ (3.19)

*and it follows that the determinant* det (*ω*) *in* Eq (3.18) *can be rewritten in the form*

$$\begin{array}{c} \text{det}\left(\omega \right)=-(\mu_{1}^{2}+\mu_{2}^{2}-\mu_{3}^{2})=-\varphi_{L}(\mu ,\mu ), \end{array}$$ (3.20)

*as stated in* Eq (3.16).

**Theorem 3.4**. *The coefficients* (2.8) *of the second fundamental form of the (timelike/spacelike) q-Bernstein-Bézier surface* (qbbs) *can be written in following form*

$$\begin{array}{c} e={\left(\sqrt{-\eta \;\varphi_{L}(\mu ,\mu )}\right)}^{-1}\varphi_{L}(\sum_{ȷ=0}^{m-2}\sum_{\ell =0}^{n}Q_{ȷ}^{m-2,q}(u)Q_{\ell }^{n,q}(v){♭}_{ȷ\ell }^{\left(2,0\right)},\mu ), \end{array}$$ (3.21)

$$\begin{array}{c} f={\left(\sqrt{-\eta \;\varphi_{L}(\mu ,\mu )}\right)}^{-1}\varphi_{L}(\sum_{ȷ=0}^{m-1}\sum_{\ell =0}^{n-1}Q_{ȷ}^{m-1,q}(u)Q_{\ell }^{n-1,q}(v){♭}_{ȷ\ell }^{\left(1,1\right)},\mu ), \end{array}$$ (3.22)

$$\begin{array}{c} g={\left(\sqrt{-\eta \;\varphi_{L}(\mu ,\mu )}\right)}^{-1}\varphi_{L}(\sum_{ȷ=0}^{m}\sum_{\ell =0}^{n-2}Q_{ȷ}^{m,q}(u)Q_{\ell }^{n-2,q}(v){♭}_{ȷ\ell }^{\left(0,2\right)},\mu ). \end{array}$$ (3.23)

*Proof*. The second fundamental coefficient $e=\varphi_{L}(s_{uu},N),f=\varphi_{L}(s_{uv},N),g=\varphi_{L}(s_{vv},N)$ of Eq (2.8) for the (timelike/spacelike) *q*-Bernstein-Bézier surface **s**(*u*, *v*) can be computed by utilizing the second-order partial derivative **s**_*uu*_(*u*, *v*), **s**_*uv*_(*u*, *v*) and **s**_*vv*_(*u*, *v*) respectively, of Eqs (2.29), (2.31) and (2.33) and the unit normal-vector field **N**, Eq (3.12). It follows that

$$\begin{array}{c} e=\frac{-\text{det}(\sum_{ȷ=0}^{m-2}\sum_{\ell =0}^{n}Q_{ȷ}^{m-2,q}(u)Q_{\ell }^{n,q}(v){♭}_{ȷ\ell }^{\left(2,0\right)},\sum_{ȷ=0}^{m-1}\sum_{\ell =0}^{n}Q_{ȷ}^{m-1,q}(u)Q_{\ell }^{n,q}(v){♭}_{ȷ\ell }^{\left(1,0\right)},\sum_{ȷ=0}^{m}\sum_{\ell =0}^{n-1}Q_{ȷ}^{m,q}(u)Q_{\ell }^{n-1,q}(v){♭}_{ȷ\ell }^{\left(0,1\right)})}{\left\|\sum_{ȷ=0}^{m-1}\sum_{\ell =0}^{n}Q_{ȷ}^{m-1,q}(u)Q_{\ell }^{n,q}(v){♭}_{ȷ\ell }^{\left(1,0\right)}\;\wedge_{L}\;\sum_{ȷ=0}^{m}\sum_{\ell =0}^{n-1}Q_{ȷ}^{m,q}(u)Q_{\ell }^{n-1,q}(v){♭}_{ȷ\ell }^{\left(0,1\right)}\right\|}, \end{array}$$ (3.24)

further simplification of Eq (3.24) results in,

$$\begin{array}{c} e=\frac{-\sum_{ȷ=0}^{m-2}\sum_{\ell =0}^{n}Q_{ȷ}^{m-2,q}(u)Q_{\ell }^{n,q}(v)x_{ȷ\ell }^{\left(2,0\right)}\left(-\mu_{1}\right)-\sum_{ȷ=0}^{m-2}\sum_{\ell =0}^{n}Q_{ȷ}^{m-2,q}(u)Q_{\ell }^{n,q}(v)y_{ȷ\ell }^{\left(2,0\right)}\left(-\mu_{2}\right)-\sum_{ȷ=0}^{m-2}\sum_{\ell =0}^{n}Q_{ȷ}^{m-2,q}(u)Q_{\ell }^{n,q}(v)x_{ȷ\ell }^{\left(2,0\right)}\mu_{3}}{\sqrt{\left|\mu_{1}^{2}+\mu_{2}^{2}-\mu_{3}^{2}\right|}}, \end{array}$$ (3.25)

and thus the Eq (3.25) can be written in the following simpler useful form

$$\begin{array}{c} e=\frac{\sum_{ȷ=0}^{m-2}\sum_{\ell =0}^{n}Q_{ȷ}^{m-2,q}(u)Q_{\ell }^{n,q}(v)x_{ȷ\ell }^{\left(2,0\right)}\mu_{1}+\sum_{ȷ=0}^{m-2}\sum_{\ell =0}^{n}Q_{ȷ}^{m-2,q}(u)Q_{\ell }^{n,q}(v)y_{ȷ\ell }^{\left(2,0\right)}\mu_{2}-\sum_{ȷ=0}^{m-2}\sum_{\ell =0}^{n}Q_{ȷ}^{m-2,q}(u)Q_{\ell }^{n,q}(v)x_{ȷ\ell }^{\left(2,0\right)}\mu_{3}}{\sqrt{-\eta (\mu_{1}^{2}+\mu_{2}^{2}-\mu_{3}^{2})}}. \end{array}$$ (3.26)

Similarly, the coefficients *f* and *g* (of Eq (2.8)) of the second-fundamental form can be found by using the second-order partial derivative **s**_*uv*_(*u*, *v*) and **s**_*vv*_(*u*, *v*) of Eqs (2.31) and (2.33) respectively, and the normal vector **N**, Eq (3.12).

**Corollary 3.4.1**. *The coefficients e, f, g* (Eqs (3.21), (3.22) *and* (3.23)) *of the second-fundamental form of the (timelike/spacelike) q-Bernstein-Bézier surface* (qbbs) *in the Minkowski space*-$R_{1}^{3}$, *at the min-point* (*u*, *v*) = (0, 0) *are*,

$$\begin{array}{c} e=\frac{\varphi_{L}({♭}_{ȷ\ell }^{\left(2,0\right)},\mu )}{\sqrt{-\eta \;\varphi_{L}(\mu ,\mu )}},\;f=\frac{\varphi_{L}({♭}_{ȷ\ell }^{\left(1,1\right)},\;\mu )}{\sqrt{-\eta \;\varphi_{L}(\mu ,\mu )}},\;g=\frac{\varphi_{L}({♭}_{ȷ\ell }^{\left(0,2\right)},\;\mu )}{\sqrt{-\eta \;\varphi_{L}(\mu ,\mu )}}, \end{array}$$ (3.27)

*where*, ***μ*** = (*μ*_1_, *μ*_2_, *μ*_3_) *and* *μ*_1_, *μ*_2_ *and* *μ*_3_ *are given by the* Eq (3.19).

**Theorem 3.5**. *We can find the Gaussian-curvature* $K=\eta (\frac{eg-f^{2}}{EG-F^{2}})$ *of the (timelike/spacelike)* *q-Bernstein-Bézier surface* **s**(*u*, *v*) *in Minkowski space by using the fundamental coefficients given in the Theorem 3.1 and the Theorem 3.4. It follows that the Gaussian-curvature K of the q-Bernstein-Bézier surface* (qbbs) *can be written as*,

$$\begin{array}{c} \begin{array}{cc} K= & \frac{\eta }{\varphi_{L}^{2}(\mu ,\mu )}[\varphi_{L}(\sum_{ȷ=0}^{m-2}\sum_{\ell =0}^{n}Q_{ȷ}^{m-2,q}(u)Q_{\ell }^{n,q}(v){♭}_{ȷ\ell }^{\left(2,0\right)},\mu )\varphi_{L}(\sum_{ȷ=0}^{m}\sum_{\ell =0}^{n-2}Q_{ȷ}^{m,q}(u)Q_{\ell }^{n-2,q}(v){♭}_{ȷ\ell }^{\left(0,2\right)},\mu )-\\ & \varphi_{L}^{2}(\sum_{ȷ=0}^{m-1}\sum_{\ell =0}^{n-1}Q_{ȷ}^{m-1,q}(u)Q_{\ell }^{n-1,q}(v){♭}_{ȷ\ell }^{\left(1,1\right)},\mu )] \end{array} \end{array}$$ (3.28)

*Similarly, we can find the mean-curvature H of the q-Bernstein-Bézier surface* (qbbs) *in the Minkowski space and it follows that*,

$$\begin{array}{c} \begin{array}{cc} H & =\frac{-\eta }{2\sqrt{\eta \varphi_{L}^{3}\left(\mu ,\mu \right)}}\times \\ & [(\sum_{ȷ,\ell =0}^{m-2,n}Q_{ȷ}^{m-2,q}\varphi_{L}(u)Q_{\ell }^{n,q}(v){♭}_{ȷ\ell }^{\left(2,0\right)},\mu )\;\varphi_{L}\;(\sum_{ȷ,\ell =0}^{m,n-1}Q_{ȷ}^{m,q}(u)Q_{\ell }^{n-1,q}(v){♭}_{ȷ\ell }^{\left(0,1\right)},\sum_{ȷ,\ell =0}^{m,n-1}Q_{ȷ}^{m,q}(u)Q_{\ell }^{n-1,q}(v){♭}_{ȷ\ell }^{\left(0,1\right)})-2\times \\ & \varphi_{L}(\sum_{ȷ,\ell =0}^{m-1,n-1}Q_{ȷ}^{m-1,q}(u)Q_{\ell }^{n-1,q}(v){♭}_{ȷ\ell }^{\left(1,1\right)},\mu )\;\varphi_{L}\;(\sum_{ȷ,\ell =0}^{m-1,n}Q_{ȷ}^{m-1,q}(u)Q_{\ell }^{n,q}(v){♭}_{ȷ\ell }^{\left(1,0\right)},\sum_{ȷ,\ell =0}^{m,n-1}Q_{ȷ}^{m,q}(u)Q_{\ell }^{n-1,q}(v){♭}_{ȷ\ell }^{\left(0,1\right)})+\\ & \varphi_{L}(\sum_{ȷ,\ell =0}^{m,n-2}Q_{ȷ}^{m,q}(u)Q_{\ell }^{n-2,q}(v){♭}_{ȷ\ell }^{\left(0,2\right)},\mu )\;\varphi_{L}\;(\sum_{ȷ,\ell =0}^{m-1,n}Q_{ȷ}^{m-1,q}(u)Q_{\ell }^{n,q}(v){♭}_{ȷ\ell }^{\left(1,0\right)},\sum_{ȷ,\ell =0}^{m-1,n}Q_{ȷ}^{m-1,q}(u)Q_{\ell }^{n,q}(v){♭}_{ȷ\ell }^{\left(1,0\right)})] \end{array} \end{array}$$ (3.29)

We may skip the proof since the computations involved are straightforward.

**Corollary 3.5.1**. *Thus, the Gaussian-curvature (from the above* Eq (3.28)) *and the mean-curvature (from the* Eq (3.29)) *of the (timelike/spacelike) q-Bernstein-Bézier surface* **s**(*u*, *v*) *in the Minkowski space*-$R_{1}^{3}$, *at the minimum-point* (*u*, *v*) = (0, 0) *come out to be*,

$$\begin{array}{c} K=\frac{-\eta }{\varphi_{L}^{2}\left(\mu ,\mu \right)}(\varphi_{L}({♭}_{00}^{\left(2,0\right)},\mu )\varphi_{L}({♭}_{00}^{\left(0,2\right)},\mu )-\varphi_{L}^{2}({♭}_{00}^{\left(1,1\right)},\mu )), \end{array}$$ (3.30)

*and*

$$\begin{array}{c} \begin{array}{cc} H= & \frac{-\eta }{2\sqrt{-\eta \varphi_{L}^{3}\left(\mu ,\mu \right)}}[\varphi_{L}\left({♭}_{00}^{\left(2,0\right)},\mu \right)\varphi_{L}\left({♭}_{00}^{\left(0,1\right)},{♭}_{00}^{\left(0,1\right)}\right)-2\varphi_{L}\left({♭}_{00}^{\left(1,1\right)},\mu \right)\times \\ & \varphi_{L}\left({♭}_{00}^{\left(1,0\right)},{♭}_{00}^{\left(0,1\right)}\right)+\varphi_{L}\left({♭}_{00}^{\left(0,2\right)},\mu \right)\varphi_{L}\left({♭}_{00}^{\left(1,0\right)},{♭}_{00}^{\left(1,0\right)}\right)]. \end{array} \end{array}$$ (3.31)

**Theorem 3.6**. *The coefficients of the matrix* $V=[\begin{array}{cc} b_{11} & b_{12}\\ b_{21} & b_{22} \end{array}]$ *corresponding to the shape-operator of the (timelike/spacelike) q-Bernstein-Bézier surface* **s**(*u*, *v*) *in Minkowski space*-$R_{1}^{3}$ *are*,

$$\begin{array}{c} \begin{array}{cc} & b_{11}=\frac{-1}{\sqrt{-\eta \;\varphi_{L}^{3}\left(\mu ,\mu \right)}}\times \\ & (\begin{array}{cc} & \varphi_{L}(\sum_{ȷ,\ell =0}^{m-2,n}Q_{ȷ}^{m-2,q}(u)Q_{\ell }^{n,q}(v){♭}_{ȷ\ell }^{\left(2,0\right)},\mu )\;\varphi_{L}(\sum_{ȷ,\ell =0}^{m,n-1}Q_{ȷ}^{m,q}(u)Q_{\ell }^{n-1,q}(v){♭}_{ȷ\ell }^{\left(0,1\right)},\sum_{ȷ,\ell =0}^{m,n-1}Q_{ȷ}^{m,q}(u)Q_{\ell }^{n-1,q}(v){♭}_{ȷ\ell }^{\left(0,1\right)})-\\ & \varphi_{L}(\sum_{ȷ,\ell =0}^{m-1,n-1}Q_{ȷ}^{m-1,q}(u)Q_{\ell }^{n-1,q}(v){♭}_{ȷ\ell }^{\left(1,1\right)},\mu )\;\varphi_{L}(\sum_{ȷ,\ell =0}^{m-1,n}Q_{ȷ}^{m-1,q}(u)Q_{\ell }^{n,q}(v){♭}_{ȷ\ell }^{\left(1,0\right)},\sum_{ȷ,\ell =0}^{m,n-1}Q_{ȷ}^{m,q}(u)Q_{\ell }^{n-1,q}(v){♭}_{ȷ\ell }^{\left(0,1\right)}) \end{array}), \end{array} \end{array}$$ (3.32)

$$\begin{array}{c} \begin{array}{cc} & b_{12}=\frac{1}{\sqrt{-\eta \;\varphi_{L}^{3}\left(\mu ,\mu \right)}}\times \\ & (\begin{array}{cc} & \varphi_{L}(\sum_{ȷ,\ell =0}^{m-1,n-1}Q_{ȷ}^{m-1,q}(u)Q_{\ell }^{n-1,q}(v){♭}_{ȷ\ell }^{\left(1,1\right)},\mu )\;\varphi_{L}(\sum_{ȷ,\ell =0}^{m,n-1}Q_{ȷ}^{m,q}(u)Q_{\ell }^{n-1,q}(v){♭}_{ȷ\ell }^{\left(0,1\right)},\sum_{ȷ,\ell =0}^{m,n-1}Q_{ȷ}^{m,q}(u)Q_{\ell }^{n-1,q}(v){♭}_{ȷ\ell }^{\left(0,1\right)})-\\ & \varphi_{L}(\sum_{ȷ,\ell =0}^{m,n-2}Q_{ȷ}^{m,q}(u)Q_{\ell }^{n-2,q}(v){♭}_{ȷ\ell }^{\left(0,2\right)},\mu )\;\varphi_{L}(\sum_{ȷ,\ell =0}^{m-1,n}Q_{ȷ}^{m,q}(u)Q_{\ell }^{n,q}(v){♭}_{ȷ\ell }^{\left(1,0\right)},\sum_{ȷ,\ell =0}^{m,n-1}Q_{ȷ}^{m,q}(u)Q_{\ell }^{n-1,q}(v){♭}_{ȷ\ell }^{\left(0,1\right)}) \end{array}), \end{array} \end{array}$$ (3.33)

$$\begin{array}{c} \begin{array}{cc} & b_{21}=\frac{1}{\sqrt{-\eta \;\varphi_{L}^{3}\left(\mu ,\mu \right)}}\times \\ & (\begin{array}{cc} & \varphi_{L}(\sum_{ȷ,\ell =0}^{m-2,n}Q_{ȷ}^{m-2,q}(u)Q_{\ell }^{n,q}(v){♭}_{ȷ\ell }^{\left(2,0\right)},\mu )\;\varphi_{L}(\sum_{ȷ,\ell =0}^{m-1,n}Q_{ȷ}^{m-1,q}(u)Q_{\ell }^{n,q}(v){♭}_{ȷ\ell }^{\left(1,0\right)},\sum_{ȷ,\ell =0}^{m,n-1}Q_{ȷ}^{m,q}(u)Q_{\ell }^{n-1,q}(v){♭}_{ȷ\ell }^{\left(0,1\right)})-\\ & \varphi_{L}(\sum_{ȷ,\ell =0}^{m-1,n-1}Q_{ȷ}^{m-1,q}(u)Q_{\ell }^{n-1,q}(v){♭}_{ȷ\ell }^{\left(1,1\right)},\mu )\;\varphi_{L}(\sum_{ȷ,\ell =0}^{m-1,n}Q_{ȷ}^{m-1,q}(u)Q_{\ell }^{n,q}(v){♭}_{ȷ\ell }^{\left(1,0\right)},\sum_{ȷ,\ell =0}^{m-1,n}Q_{ȷ}^{m-1,q}(u)Q_{\ell }^{n,q}(v){♭}_{ȷ\ell }^{\left(1,0\right)}) \end{array}), \end{array} \end{array}$$ (3.34)

*and*,

$$\begin{array}{c} \begin{array}{cc} & b_{22}=\frac{1}{\sqrt{-\eta \;\varphi_{L}^{3}\left(\mu ,\mu \right)}}\times \\ & (\begin{array}{cc} & \varphi_{L}(\sum_{ȷ,\ell =0}^{m-1,n-1}Q_{ȷ}^{m-1,q}(u)Q_{\ell }^{n-1,q}(v){♭}_{ȷ\ell }^{\left(1,1\right)},\mu )\;\varphi_{L}(\sum_{ȷ,\ell =0}^{m-1,n}Q_{ȷ}^{m-1,q}(u)Q_{\ell }^{n,q}(v){♭}_{ȷ\ell }^{\left(1,0\right)},\sum_{ȷ,\ell =0}^{m,n-1}Q_{ȷ}^{m,q}(u)Q_{\ell }^{n-1,q}(v){♭}_{ȷ\ell }^{\left(0,1\right)})-\\ & \varphi_{L}(\sum_{ȷ,\ell =0}^{m,n-2}Q_{ȷ}^{m,q}(u)Q_{\ell }^{n-2,q}(v){♭}_{ȷ\ell }^{\left(0,2\right)},\mu )\;\varphi_{L}(\sum_{ȷ,\ell =0}^{m-1,n}Q_{ȷ}^{m-1,q}(u)Q_{\ell }^{n,q}(v){♭}_{ȷ\ell }^{\left(1,0\right)},\sum_{ȷ,\ell =0}^{m-1,n}Q_{ȷ}^{m-1,q}(u)Q_{\ell }^{n,q}(v){♭}_{ȷ\ell }^{\left(1,0\right)}) \end{array}). \end{array} \end{array}$$ (3.35)

*Proof*. The matrix corresponding to the shape-operator of the (timelike/spacelike) *q*-Bernstein-Bézier surface (qbbs) in Minkowski space-$R_{1}^{3}$ is

$$\begin{array}{c} V=(\begin{array}{cc} b_{11} & b_{12}\\ b_{21} & b_{22} \end{array})=\frac{1}{EG-F^{2}}[\begin{array}{cc} G & -F\\ -F & E \end{array}][\begin{array}{ccc} e & & f\\ f & & g \end{array}]. \end{array}$$ (3.36)

Note that the fundamental coefficients are given in the statements of the Theorem 3.1 and Theorem 3.4. Thus, the matrix element $b_{11}=\frac{e\;G-f\;F}{E\;G-F^{2}}$ of the above matrix *V* is

$$\begin{array}{c} \begin{array}{cc} b_{11} & =\frac{-1}{\sqrt{-\eta \varphi_{L}^{3}\left(\mu ,\mu \right)}}\times \\ & [\varphi_{L}(\sum_{ȷ,\ell =0}^{m-2,n}Q_{ȷ}^{m-2,q}(u)Q_{\ell }^{n,q}(v){♭}_{ȷ\ell }^{\left(2,0\right)},\mu )\varphi_{L}(\sum_{ȷ,\ell =0}^{m,n-1}Q_{ȷ}^{m,q}(u)Q_{\ell }^{n-1,q}(v){♭}_{ȷ\ell }^{\left(0,1\right)},\sum_{ȷ,\ell =0}^{m,n-1}Q_{ȷ}^{m,q}(u)Q_{\ell }^{n-1,q}(v){♭}_{ȷ\ell }^{\left(0,1\right)})+\\ & \varphi_{L}(\sum_{ȷ,\ell =0}^{m-1,n-1}Q_{ȷ}^{m-1,q}(u)Q_{\ell }^{n-1,q}(v){♭}_{ȷ\ell }^{\left(1,1\right)},\mu )\varphi_{L}(\sum_{ȷ,\ell =0}^{m-1,n}Q_{ȷ}^{m-1,q}(u)Q_{\ell }^{n,q}(v){♭}_{ȷ\ell }^{\left(1,0\right)},\sum_{ȷ,\ell =0}^{m,n-1}Q_{ȷ}^{m,q}(u)Q_{\ell }^{n-1,q}(v){♭}_{ȷ\ell }^{\left(0,1\right)})]. \end{array} \end{array}$$ (3.37)

Further simplification in the above Eq (3.37) reduces it to the Eq (3.32). In the similar way, we can compute the rest of the matrix elements, *b*_12_, *b*_21_ and *b*_22_ as given in Eqs (3.33)–(3.35) of the matrix *V* (3.36).

**Theorem 3.7**. *We can adopt the alternative approach for convenience, in order to find the Gaussian-curvature and the mean-curvature of the (timelike/spacelike) q-Bernstein-Bézier surface* **s**(*u*, *v*) *by using the shape-operator of the surface in Minkowski space*-$R_{1}^{3}$, *they are*

$$\begin{array}{c} \begin{array}{cc} K= & -\frac{\eta }{\varphi_{L}^{2}\left(\mu ,\mu \right)}[\varphi_{L}(\sum_{ȷ,\ell =0}^{m-2,n}Q_{ȷ}^{m-2,q}(u)Q_{\ell }^{n,q}(v){♭}_{ȷ\ell }^{\left(2,0\right)},\mu )\varphi_{L}(\sum_{ȷ,\ell =0}^{m,n-2}Q_{ȷ}^{m,q}(u)Q_{\ell }^{n-2,q}(v){♭}_{ȷ\ell }^{\left(0,2\right)},\mu )-\\ & \;\varphi_{L}{\left(\sum_{ȷ,\ell =0}^{m-1,n-1}Q_{ȷ}^{m-1,q}(u)Q_{\ell }^{n-1,q}(v){♭}_{ȷ\ell }^{\left(1,1\right)},\mu \right)}^{2}] \end{array}, \end{array}$$ (3.38)

and

$$\begin{array}{c} \begin{array}{cc} & H=-\frac{\eta }{2\sqrt{\eta \;\varphi_{L}^{3}\left(\mu ,\mu \right)}}\times \\ & \begin{array}{cc} & [\varphi_{L}(\sum_{ȷ,\ell =0}^{m-2,n}Q_{ȷ}^{m-2,q}(u)Q_{\ell }^{n,q}(v){♭}_{ȷ\ell }^{\left(2,0\right)},\mu )\;\varphi_{L}(\sum_{ȷ,\ell =0}^{m,n-1}Q_{ȷ}^{m,q}(u)Q_{\ell }^{n-1,q}(v){♭}_{ȷ\ell }^{\left(0,1\right)},\sum_{ȷ,\ell =0}^{m,n-1}Q_{ȷ}^{m,q}(u)Q_{\ell }^{n-1,q}(v){♭}_{ȷ\ell }^{\left(0,1\right)})-2\times \\ & \varphi_{L}(\sum_{ȷ,\ell =0}^{m-1,n-1}Q_{ȷ}^{m-1,q}(u)Q_{\ell }^{n-1,q}(v){♭}_{ȷ\ell }^{\left(1,1\right)},\mu )\;\varphi_{L}(\sum_{ȷ,\ell =0}^{m-1,n}Q_{ȷ}^{m-1,q}(u)Q_{\ell }^{n,q}(v){♭}_{ȷ\ell }^{\left(1,0\right)},\sum_{ȷ,\ell =0}^{m,n-1}Q_{ȷ}^{m,q}(u)Q_{\ell }^{n-1,q}(v){♭}_{ȷ\ell }^{\left(0,1\right)})+\\ & \varphi_{L}(\sum_{ȷ,\ell =0}^{m,n-2}Q_{ȷ}^{m,q}(u)Q_{\ell }^{n-2,q}(v){♭}_{ȷ\ell }^{\left(0,2\right)},\mu )\;\varphi_{L}(\sum_{ȷ,\ell =0}^{m-1,n}Q_{ȷ}^{m-1,q}(u)Q_{\ell }^{n,q}(v){♭}_{ȷ\ell }^{\left(1,0\right)},\sum_{ȷ,\ell =0}^{m-1,n}Q_{ȷ}^{m-1,q}(u)Q_{\ell }^{n,q}(v){♭}_{ȷ\ell }^{\left(1,0\right)})] \end{array}. \end{array} \end{array}$$ (3.39)

*Proof*. As mentioned above in the statement that the Gaussian-curvature and mean-curvature of the (timelike/spacelike) *q*-Bernstein-Bézier surface **s**(*u*, *v*) in Minkowski space-$R_{1}^{3}$ can be found by using the matrix-coefficients corresponding to the shape-operator. The Gaussian-curvature (*K* = *η* det (*V*) = *η*(*b*_11_*b*_22_ − *b*_12_*b*_21_)) of the (timelike/spacelike) *q*-Bernstein-Bézier surface (qbbs), by virtue of the shape operator matrix coefficients (3.32)–(3.35)), turns up

$$\begin{array}{c} K=\frac{\eta (\mu_{3}^{2}-\mu_{2}^{2}-\mu_{1}^{2})}{\varphi_{L}^{3}(\mu ,\mu )}(\begin{array}{cc} & \varphi_{L}(\sum_{ȷ,\ell =0}^{m-2,n}Q_{ȷ}^{m-2,q}(u)Q_{\ell }^{n,q}(v){♭}_{ȷ\ell }^{\left(2,0\right)},\mu )\varphi_{L}(\sum_{ȷ,\ell =0}^{m,n-2}Q_{ȷ}^{m,q}(u)Q_{\ell }^{n-2,q}(v){♭}_{ȷ\ell }^{\left(0,2\right)},\mu )-\\ & \varphi_{L}{\left(\sum_{ȷ,\ell =0}^{m-1,n-1}Q_{ȷ}^{m-1,q}(u)Q_{\ell }^{n-1,q}(v){♭}_{ȷ\ell }^{\left(1,1\right)},\mu \right)}^{2} \end{array}). \end{array}$$ (3.40)

Plugging the Eq (3.20) in above Eq (3.40) for the Gaussian curvature *K* in the following form

$$\begin{array}{c} \begin{array}{cc} & K=\frac{-\eta \;\varphi_{L}(\mu ,\mu )}{\varphi_{L}^{3}(\mu ,\mu )}(\begin{array}{cc} & \varphi_{L}(\sum_{ȷ,\ell =0}^{m-2,n}Q_{ȷ}^{m-2,q}(u)Q_{\ell }^{n,q}(v){♭}_{ȷ\ell }^{\left(2,0\right)},\mu )\varphi_{L}(\sum_{ȷ,\ell =0}^{m,n-2}Q_{ȷ}^{m,q}(u)Q_{\ell }^{n-2,q}(v){♭}_{ȷ\ell }^{\left(0,2\right)},\mu )-\\ & \varphi_{L}{\left(\sum_{ȷ,\ell =0}^{m-1,n-1}Q_{ȷ}^{m-1,q}(u)Q_{\ell }^{n-1,q}(v){♭}_{ȷ\ell }^{\left(1,1\right)},\mu \right)}^{2} \end{array}), \end{array} \end{array}$$ (3.41)

a little simplification in above equation, reduces it to

$$\begin{array}{c} \begin{array}{cc} & K=\frac{-\eta }{\varphi_{L}^{2}(\mu ,\mu )}(\begin{array}{cc} & \varphi_{L}(\sum_{ȷ,\ell =0}^{m-2,n}Q_{ȷ}^{m-2,q}(u)Q_{\ell }^{n,q}(v){♭}_{ȷ\ell }^{\left(2,0\right)},\mu )\varphi_{L}(\sum_{ȷ,\ell =0}^{m,n-2}Q_{ȷ}^{m,q}(u)Q_{\ell }^{n-2,q}(v){♭}_{ȷ\ell }^{\left(0,2\right)},\mu )-\\ & \varphi_{L}{\left(\sum_{ȷ,\ell =0}^{m-1,n-1}Q_{ȷ}^{m-1,q}(u)Q_{\ell }^{n-1,q}(v){♭}_{ȷ\ell }^{\left(1,1\right)},\mu \right)}^{2} \end{array}). \end{array} \end{array}$$ (3.42)

Similarly, the mean curvature, $H=\frac{\eta }{2}\text{tr}\left(V\right)=\frac{\eta }{2}(b_{11}+b_{22})$, can be obtained by utilizing the shape operator matrix coefficients (3.32)–(3.35) and it takes the form,

$$\begin{array}{c} \begin{array}{cc} & H=\frac{-\eta }{2\sqrt{-\eta \varphi_{L}^{3}\left(\mu ,\mu \right)}}\varphi_{L}(\sum_{ȷ,\ell =0}^{m-2,n}Q_{ȷ}^{m-2,q}(u)Q_{\ell }^{n,q}(v){♭}_{ȷ\ell }^{\left(2,0\right)},\mu )\varphi_{L}(\sum_{ȷ,\ell =0}^{m,n-1}Q_{ȷ}^{m,q}(u)Q_{\ell }^{n-1,q}(v){♭}_{ȷ\ell }^{\left(0,1\right)},\sum_{ȷ,\ell =0}^{m,n-1}Q_{ȷ}^{m,q}(u)Q_{\ell }^{n-1,q}(v){♭}_{ȷ\ell }^{\left(0,1\right)})\\ & +\frac{\eta }{2\sqrt{-\eta \varphi_{L}^{3}\left(\mu ,\mu \right)}}\varphi_{L}(\sum_{ȷ,\ell =0}^{m-1,n-1}Q_{ȷ}^{m-1,q}(u)Q_{\ell }^{n-1,q}(v){♭}_{ȷ\ell }^{\left(1,1\right)},\mu )\varphi_{L}(\sum_{ȷ,\ell =0}^{m-1,n}Q_{ȷ}^{m-1,q}(u)Q_{\ell }^{n,q}(v){♭}_{ȷ\ell }^{\left(1,0\right)},\sum_{ȷ,\ell =0}^{m,n-1}Q_{ȷ}^{m,q}(u)Q_{\ell }^{n-1,q}(v){♭}_{ȷ\ell }^{\left(0,1\right)})\\ & +\frac{\eta }{2\sqrt{-\eta \varphi_{L}^{3}\left(\mu ,\mu \right)}}\varphi_{L}(\sum_{ȷ,\ell =0}^{m-1,n-1}Q_{ȷ}^{m-1,q}(u)Q_{\ell }^{n-1,q}(v){♭}_{ȷ\ell }^{\left(1,1\right)},\mu )\varphi_{L}(\sum_{ȷ,\ell =0}^{m-1,n}Q_{ȷ}^{m-1,q}(u)Q_{\ell }^{n,q}(v){♭}_{ȷ\ell }^{\left(1,0\right)},\sum_{ȷ,\ell =0}^{m,n-1}Q_{ȷ}^{m,q}(u)Q_{\ell }^{n-1,q}(v){♭}_{ȷ\ell }^{\left(0,1\right)})\\ & +\frac{-\eta }{2\sqrt{-\eta \varphi_{L}^{3}\left(\mu ,\mu \right)}}\varphi_{L}(\sum_{ȷ,\ell =0}^{m,n-2}Q_{ȷ}^{m,q}(u)Q_{\ell }^{n-2,q}(v){♭}_{ȷ\ell }^{\left(0,2\right)},\mu )\varphi_{L}(\sum_{ȷ,\ell =0}^{m-1,n}Q_{ȷ}^{m-1,q}(u)Q_{\ell }^{n,q}(v){♭}_{ȷ\ell }^{\left(1,0\right)},\sum_{ȷ,\ell =0}^{m-1,n}Q_{ȷ}^{m-1,q}(u)Q_{\ell }^{n,q}(v){♭}_{ȷ\ell }^{\left(1,0\right)}). \end{array} \end{array}$$ (3.43)

**Corollary 3.7.1**. *The matrix-coefficient* *b*_11_ (Eq (3.32)) *of the shape operator matrix V of the (timelike/spacelike) q-Bernstein-Bézier surface* **s**(*u*, *v*) *in the Minkowski space*-$R_{1}^{3}$, *at the point* (*u*, *v*) = (0, 0) *turns up*,

$$\begin{array}{c} b_{11}=\frac{-1}{\sqrt{-\eta \;\varphi_{L}^{3}(\mu ,\mu )}}(\varphi_{L}({♭}_{00}^{\left(2,0\right)},\mu )\;\varphi_{L}({♭}_{00}^{\left(0,1\right)},{♭}_{00}^{\left(0,1\right)})-\varphi_{L}({♭}_{00}^{\left(1,1\right)},\mu )\varphi_{L}({♭}_{00}^{\left(1,0\right)},{♭}_{00}^{\left(0,1\right)})). \end{array}$$ (3.44)

*In the similar manner, we can write the shape operator matrix coefficient b*_12_ (Eq (3.33)) *of the matrix V in Minkowski space*-$R_{1}^{3}$ *at the point* (*u*, *v*) = (0, 0), *and it appears as*,

$$\begin{array}{c} b_{12}=\frac{-1}{\sqrt{-\eta \;\varphi_{L}^{3}(\mu ,\mu )}}(\varphi_{L}({♭}_{00}^{\left(1,1\right)},\mu )\;\varphi_{L}({♭}_{00}^{\left(0,1\right)},{♭}_{00}^{\left(0,1\right)})-\varphi_{L}({♭}_{00}^{\left(0,2\right)},\mu )\varphi_{L}({♭}_{00}^{\left(1,0\right)},{♭}_{00}^{\left(0,1\right)})). \end{array}$$ (3.45)

*Similarly the shape operator matrix coefficients* *b*_21_ and *b*_22_ (Eqs (3.34) *and* (3.35)) *of the matrix V of the surface* **s**(*u*, *v*) *in Minkowski space*-$R_{1}^{3}$, *at the point* (*u*, *v*) = (0, 0) *are*,

$$\begin{array}{c} b_{21}=\frac{1}{\sqrt{-\eta \;\varphi_{L}^{3}(\mu ,\mu )}}(\varphi_{L}({♭}_{00}^{\left(2,0\right)},\mu )\;\varphi_{L}({♭}_{00}^{\left(1,0\right)},{♭}_{00}^{\left(0,1\right)})-\varphi_{L}({♭}_{00}^{\left(1,1\right)},\mu )\varphi_{L}({♭}_{00}^{\left(1,0\right)},{♭}_{00}^{\left(1,0\right)})). \end{array}$$ (3.46)

$$\begin{array}{c} b_{22}=\frac{1}{\sqrt{-\eta \;\varphi_{L}^{3}(\mu ,\mu )}}(\varphi_{L}({♭}_{00}^{\left(1,1\right)},\mu )\;\varphi_{L}({♭}_{00}^{\left(1,0\right)},{♭}_{00}^{\left(0,1\right)})-\varphi_{L}({♭}_{00}^{\left(0,2\right)},\mu )\varphi_{L}({♭}_{00}^{\left(1,0\right)},{♭}_{00}^{\left(0,1\right)})). \end{array}$$ (3.47)

**Theorem 3.8**. *The shape-operator matrix-coefficients of the matrix V of the (timelike/spacelike) q-Bernstein-Bézier surface* **s**(*u*, *v*) *can be used to find the Gaussian-curvature in Minkowski space*-$R_{1}^{3}$ *at the min-point* (*u*, *v*) = (0, 0). *It follows that*

$$\begin{array}{c} K=\frac{-\eta [\varphi_{L}({♭}_{00}^{\left(2,0\right)},\mu )\;\varphi_{L}({♭}_{00}^{\left(0,2\right)},\mu )-\varphi_{L}^{2}({♭}_{00}^{\left(1,1\right)},\mu )]}{\varphi_{L}^{2}(\mu ,\mu )}. \end{array}$$ (3.48)

*Similarly, we can find the mean-curvature of q-Bernstein-Bézier surface* **s**(*u*, *v*) *in Minkowski space*-$R_{1}^{3}$ *at the min-point* (*u*, *v*) = (0, 0) *and it is*,

$$\begin{array}{c} \begin{array}{cc} H= & \frac{-\eta }{2\sqrt{-\eta \varphi_{L}^{3}(\mu ,\mu )}}[\varphi_{L}({♭}_{00}^{\left(2,0\right)},\mu )\varphi_{L}({♭}_{00}^{\left(0,1\right)},{♭}_{00}^{\left(0,1\right)})-2\varphi_{L}({♭}_{00}^{\left(1,1\right)},\mu )\times \\ & \varphi_{L}({♭}_{00}^{\left(1,0\right)},{♭}_{00}^{\left(0,1\right)})+\varphi_{L}({♭}_{00}^{\left(0,2\right)},\mu )\varphi_{L}({♭}_{00}^{\left(1,0\right)},{♭}_{00}^{\left(1,0\right)})]. \end{array} \end{array}$$ (3.49)

*Proof*. The Gaussian-curvature and the mean-curvature at the minimum-point (*u*, *v*) = (0, 0) of the timelike and the spacelike-surface *q*-Bernstein-Bézier surface **s**(*u*, *v*) are calculated by substituting the coefficient values in the Corollary 3.7.1 into the Eqs (3.38) and (3.39)).

## 4 The numeric examples of (Timelike/Spacelike) *q*-Bernstein-Bézier surfaces

In this section, the shape operator dependence of timelike and spacelike *q*-Bernstein-Bézier surfaces (qbbs) in the Minkowski space-$R_{1}^{3}$ discussed in the above section 3 is implemented for the biquadratic and bicubic (timelike/spacelike) *q*-Bernstein-Bézier surfaces (qbbs). These surfaces serve as the illustrative examples as the timelike and spacelike surfaces for different values of the shape controlling parameter *q*.

*Example* 4.1. **Shape-operator of Biquadratic Timelike** *q*-**Bernstein-Bézier Surface**

Note that the *q*-Bernstein-Bézier surface **s**(*u*, *v*) (2.20) in Minkowski space-$R_{1}^{3}$ for *m* = 2, *n* = 2 reduces to the biquadratic timelike *q*-Bernstein-Bézier surface (qbbs),

$$\begin{array}{c} s\left(u,v\right)=\sum_{ȷ,\ell =0}^{2,2}Q_{ȷ}^{2,q}\left(u\right)Q_{\ell }^{2,q}\left(v\right){♭}_{ȷ\ell },\;\left(u,v\right)\in \left[0,1\right]\times \left[0,1\right]. \end{array}$$ (4.1)

The control points ${♭}_{ȷ\ell }^{\left(1,0\right)}$ and ${♭}_{ȷ\ell }^{\left(0,1\right)}$ can be computed from Eqs (2.24) and (2.26) for *ȷ*, *ℓ* = 0, 1 at the minimum point (*u*, *v*) = (0, 0) and they are,

$$\begin{array}{c} \begin{array}{cc} & {♭}_{00}^{\left(1,0\right)}=(-4,-8,-20),\;{♭}_{01}^{\left(1,0\right)}=(-4,-8,-8),\;{♭}_{10}^{\left(1,0\right)}=(-4,0,8),\\ & {♭}_{00}^{\left(0,1\right)}=(0,-12,-12),\;{♭}_{01}^{\left(0,1\right)}=(0,-8,-4),\;{♭}_{10}^{\left(0,1\right)}=(0,-12,0). \end{array} \end{array}$$ (4.2)

From Corollary 3.1.1 and the Eq (4.2), it turns out that the metric coefficients of biquadratic timelike *q*-Bernstein Bézier surface (qbbs) in Minkowski space-$R_{1}^{3}$ are,

$$\begin{array}{c} E=-320,\;F=-144,\;G=0, \end{array}$$ (4.3)

and thus the corresponding metric of the biquadratic timelike *q*-Bernstein-Bézier surface in Minkowski space-$R_{1}^{3}$ is,

$$\begin{array}{c} ds^{2}=E\;du^{2}-2F\;du\;dv+G\;dv^{2}=-320\;du^{2}-288\;du\;dv. \end{array}$$ (4.4)

Now, from the Eqs (2.35)–(2.37), the second-order partial derivatives of the biquadratic timelike *q*-Bernstein-Bézier surface **s**(*u*, *v*) in Minkowski space-$R_{1}^{3}$ are

$$\begin{array}{c} s_{uu}\left(0,0\right)=(0,-16,-56),\;s_{uv}\left(0,0\right)=(0,0,-48),\;s_{vv}\left(0,0\right)=(0,-8,-16). \end{array}$$ (4.5)

The unit normal **N** (Eq (3.15) of the Corollary 3.2.1) of the biquadratic timelike *q*-Bernstein-Bézier surface **s**(*u*, *v*) in Minkowski space-$R_{1}^{3}$ can be computed by utilizing Eq (4.2), so that

$$\begin{array}{c} N\left(u,v\right)\;{|}_{\left(u,v)\;=\;(0,0\right)}=N\left(0,0\right)=\frac{{♭}_{00}^{\left(1,0\right)}\;\wedge_{L}\;{♭}_{00}^{\left(0,1\right)}}{\left\|{♭}_{00}^{\left(1,0\right)}\;\wedge_{L}\;{♭}^{\left(0,1\right)}\right\|}=\frac{1}{144}(144,48,48). \end{array}$$ (4.6)

In this case, from the above Eq (4.6), for the Minkowski-metric it follows that,

$$\begin{array}{c} \varphi_{L}(N(0,0),N(0,0))=\eta =1>0. \end{array}$$ (4.7)

Therefore, the normal-vector **N**(*u*, *v*) is spacelike-vector (by virtue of Eq (2.2)). From the Eqs (4.5), (4.6) and (4.7), the fundamental coefficients *e*, *f*, *g* (3.27) of the biquadratic timelike *q*-Bernstein-Bézier surface (qbbs) are

$$\begin{array}{c} e=\frac{40}{3},\;f=16,\;g=\frac{8}{3}. \end{array}$$ (4.8)

Plugging the values of fundamental coefficients from Eq (4.3) in det (*ω*) (Eq (2.5)), we find that

$$\begin{array}{c} \text{det}(\omega )=E\;G-F^{2}=-{\left(144\right)}^{2}<0. \end{array}$$ (4.9)

Now, the coefficients *b*_11_, *b*_12_, *b*_21_ and *b*_22_ (by virtue of the Eqs (4.3) and (4.9)) of the matrix *V* corresponding to the shape-operator of the biquadratic timelike *q*-Bernstein-Bézier surface (qbbs) are,

$$\begin{array}{c} b_{11}=-\frac{1}{9},\;b_{12}=\frac{25}{162},\;b_{21}=-\frac{1}{54},\;b_{22}=\frac{17}{243}. \end{array}$$ (4.10)

For the biquadratic timelike, *q*-Bernstein-Bézier surface **s**(*u*, *v*) for *η* = 1, we can now find the mean and Gauss curvatures by exploiting the shape operator matrix coefficients of Eq (4.10),

$$\begin{array}{c} K=\frac{31}{2916},\;H=-\frac{22}{243}. \end{array}$$ (4.11)

**Fig 3:**
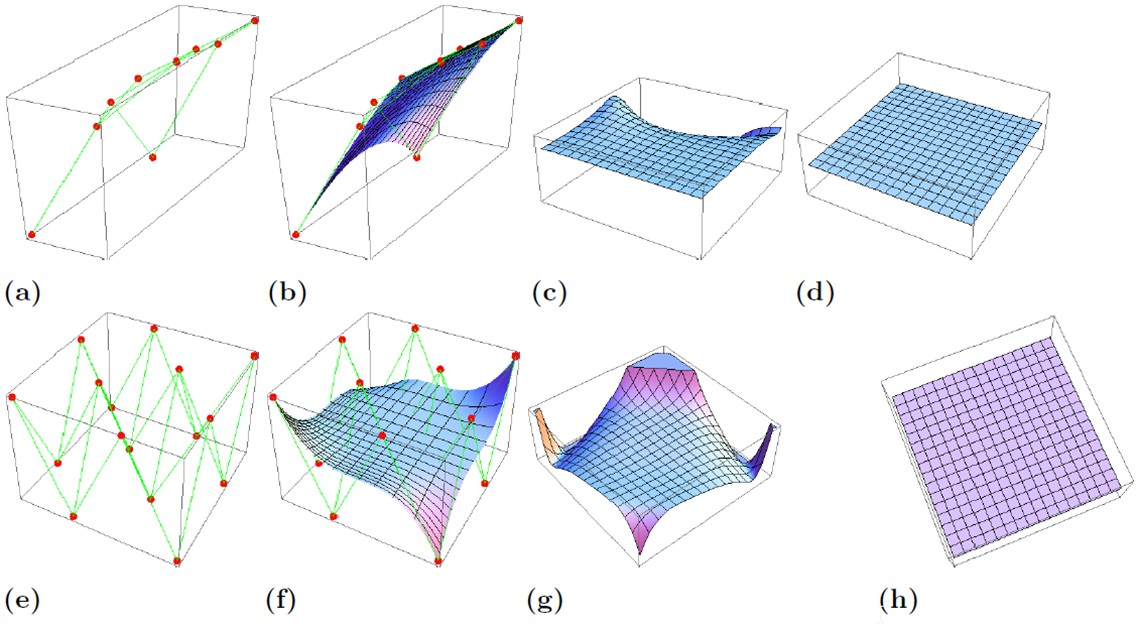
Figures (a)-(d) and (e)-(h) showcase the prescribed boundary, timelike *q*-Bernstein-Bézier surfaces for the biquadratic and bicubic cases respectively, along with their respective mean curvature at *q* = 0.2 and *q* = 1.

For the better geometric visualization of the surface, the prescribed boundary along with biquadratic timelike *q*-Bernstein-Bézier surface **s**(*u*, *v*) and the mean curvature for *q* = 0.2, 1 is shown in Fig 3.

*Example* 4.2. **Shape-operator of Bicubic Timelike** *q*-**Bernstein-Bézier Surface**

In the Minkowski space-$R_{1}^{3}$, we obtain the bicubic *q*-Bernstein-Bézier surface **s**(*u*, *v*) from (2.20) for *m* = 3, *n* = 3

$$\begin{array}{c} s\left(u,v\right)=\sum_{ȷ,\ell =0}^{3,3}Q_{ȷ}^{3,q}\left(u\right)Q_{\ell }^{3,q}\left(v\right){♭}_{ȷ\ell },\;\left(u,v\right)\in \left[0,1\right]\times \left[0,1\right]. \end{array}$$ (4.12)

The control points ${♭}_{ȷ\ell }^{\left(1,0\right)}$ and ${♭}_{ȷ\ell }^{\left(0,1\right)}$ (*ȷ*, *ℓ* = 0, 1) at the point (*u*, *v*) = (0, 0), of the bicubic timelike *q*-Bernstein-Bézier surface (Eq (4.12)) can be obtained from the Eqs (2.24) and (2.26) and they are

$$\begin{array}{c} \begin{array}{cc} & {♭}_{00}^{\left(1,0\right)}=(-6,0,12),\;{♭}_{01}^{\left(1,0\right)}=(-6,0,-12),\;{♭}_{10}^{\left(1,0\right)}=(-6,0,-12),\\ & {♭}_{00}^{\left(0,1\right)}=(0,-6,12),\;{♭}_{01}^{\left(0,1\right)}=(0,-6,-12),\;{♭}_{10}^{\left(0,1\right)}=(0,-6,-12). \end{array} \end{array}$$ (4.13)

Plugging the Eq (4.13) in the Eq (3.4) of Corollary 3.1.1, we find the metric coefficients of the *q*-Bernstein-Bézier surface (qbbs) for its bicubic timelike case in Minkowski space-$R_{1}^{3}$,

$$\begin{array}{c} E=-108,\;F=-144,\;G=-108. \end{array}$$ (4.14)

and thus the corresponding metric of bicubic timelike *q*-Bernstein-Bézier surface **s**(*u*, *v*) in Minkowski space-$R_{1}^{3}$ from the Eq (4.14) is,

$$\begin{array}{c} ds^{2}=E\;du^{2}-2F\;du\;dv+G\;dv^{2}=-108\;du^{2}+288\;du\;dv-108\;dv^{2}. \end{array}$$ (4.15)

The outcomes of the Eqs (2.35)–(2.37) enables us to find the second-order partial derivatives at the point (*u*, *v*) = (0, 0) that of the bicubic timelike *q*-Bernstein-Bézier surface **s**(*u*, *v*) in Minkowski space-$R_{1}^{3}$

$$\begin{array}{c} s_{uu}\left(0,0\right)=(0,0,97),\;s_{uv}\left(0,0\right)=(0,0,145),\;s_{vv}\left(0,0\right)=(0,0,97). \end{array}$$ (4.16)

By substituting the shape operator coefficients ${♭}_{ȷ\ell }^{\left(1,0\right)}$ and ${♭}_{ȷ\ell }^{\left(0,1\right)}$ (*ȷ*, *ℓ* = 0, 1) given by Eq (4.13) in the Eq (3.15), we find the unit normal **N** (Eq (3.15) at the point (*u*, *v*) = (0, 0), as follows

$$\begin{array}{c} N\left(0,0\right)=\frac{1}{\sqrt{7}}(-2,2,1). \end{array}$$ (4.17)

By virtue of the Eq (2.1), for the Minkowski metric, we find the norm of the unit normal **N** (from the above Eq (4.17)),

$$\begin{array}{c} \varphi_{L}(N(0,0),N(0,0))={\left(\frac{2}{\sqrt{7}}\right)}^{2}+{\left(\frac{2}{\sqrt{7}}\right)}^{2}-{\left(\frac{1}{\sqrt{7}}\right)}^{2}=\eta =1>0, \end{array}$$ (4.18)

thus the normal-vector **N**(*u*, *v*) is spacelike-vector. From the Eqs (4.16), (4.17) and (4.18), the fundamental coefficients *e*, *f*, *g*, of bicubic timelike, *q*-Bernstein-Bézier surface (qbbs) are,

$$\begin{array}{c} e=-\frac{97}{\sqrt{7}},\;f=-\frac{145}{\sqrt{7}},\;g=-\frac{97}{\sqrt{7}}. \end{array}$$ (4.19)

Plugging the values of fundamental coefficients from Eq (4.14) in det (*ω*) (Eq (2.5)), we find that

$$\begin{array}{c} \text{det}(\omega )=E\;G-F^{2}=-9072<0. \end{array}$$ (4.20)

For bicubic timelike, the coefficients *b*_11_, *b*_12_, *b*_21_ and *b*_22_ of the matrix *V* of the shape-operator (using Eqs (4.14) and (4.20)) of *q*-Bernstein-Bézier surface (qbbs) are,

$$\begin{array}{c} b_{11}=\frac{289}{252\sqrt{7}},\;b_{12}=\frac{-47}{252\sqrt{7}},\;b_{21}=\frac{-47}{252\sqrt{7}},\;b_{22}=\frac{289}{252\sqrt{7}}. \end{array}$$ (4.21)

By using the above shape operator matrix coefficients (4.21), the Gaussian-curvature and the mean-curvature of the bicubic timelike *q*-Bernstein-Bézier surface **s**(*u*, *v*) for *η* = 1, come up,

$$\begin{array}{c} K=\frac{242}{1323},\;H=\frac{289}{252\sqrt{7}}. \end{array}$$ (4.22)

The Fig 3(e), 3(f), 3(g) and 3(h) represent the prescribed boundary points and the bicubic timelike *q*-Bernstein-Bézier surface **s**(*u*, *v*), the corresponding mean curvature for *q* = 0.2 and the mean curvature for *q* = 1.

*Example* 4.3. **Shape-operator of Biquadratic Spacelike** *q*-**Bernstein-Bézier Surface**

For the biquadratic *q*-Bernstein-Bézier surface Eq (4.1), we find the control points, ${♭}_{ȷ\ell }^{\left(1,0\right)}$ and ${♭}_{ȷ\ell }^{\left(0,1\right)}$ from Eqs (2.24) and (2.26) for *ȷ*, *ℓ* = 0, 1, for spacelike case at the point (*u*, *v*) = (0, 0),

$$\begin{array}{c} \begin{array}{cc} & {♭}_{00}^{\left(1,0\right)}=(-4,0,0),\;{♭}_{01}^{\left(1,0\right)}=(-4,0,-4),\;{♭}_{10}^{\left(1,0\right)}=(-4,0,0),\\ & {♭}_{00}^{\left(0,1\right)}=(0,-4,0),\;{♭}_{01}^{\left(0,1\right)}=(0,-4,0),\;{♭}_{10}^{\left(0,1\right)}=(0,-4,-4). \end{array} \end{array}$$ (4.23)

The coefficients of the metric for the biquadratic spacelike *q*-Bernstein-Bézier surface (qbbs) (Eq (3.4) of Corollary 3.1.1) in Minkowski space-$R_{1}^{3}$ are,

$$\begin{array}{c} E=16,\;F=0,\;G=16, \end{array}$$ (4.24)

and thus the corresponding metric of the biquadratic spacelike *q*-Bernstein-Bézier surface in Minkowski space-$R_{1}^{3}$ is,

$$\begin{array}{c} ds^{2}=E\;du^{2}-2F\;du\;dv+G\;dv^{2}=16\;du^{2}+16\;dv^{2}. \end{array}$$ (4.25)

The second-order partial derivatives given by Eqs (2.35)–(2.37) of the biquadratic *q*-Bernstein-Bézier surface (qbbs) in Minkowski space-$R_{1}^{3}$ are

$$\begin{array}{c} \begin{array}{cc} & s_{uu}\left(0,0\right)=(0,0,0),\;s_{uv}\left(0,0\right)=(0,0,16),\;s_{vv}\left(0,0\right)=(0,0,0). \end{array} \end{array}$$ (4.26)

The unit normal **N** (Eq (3.15) of the Corollary 3.2.1) to the biquadratic spacelike *q*-Bernstein-Bézier surface **s**(*u*, *v*) in Minkowski space-$R_{1}^{3}$ can be computed by utilizing Eq (4.23), so that

$$\begin{array}{c} N\left(0,0\right)=(0,0,1), \end{array}$$ (4.27)

and in this case, it appears that,

$$\begin{array}{c} \varphi_{L}(N(0,0),N(0,0))=\eta =-1<0, \end{array}$$ (4.28)

and thus the normal-vector **N**(*u*, *v*) is timelike-vector. From the Eqs (4.26), (4.27) and (4.28), the fundamental coefficients *e*, *f*, *g* of the biquadratic spacelike *q*-Bernstein-Bézier surface **s**(*u*, *v*) are

$$\begin{array}{c} \begin{array}{cc} & e=0,\;f=16,\;g=0. \end{array} \end{array}$$ (4.29)

Substituting the values of fundamental coefficients from Eq (4.24) in det (*ω*) (Eq (2.5)), we find that

$$\begin{array}{c} \text{det}(\omega )=E\;G-F^{2}={\left(16\right)}^{2}>0. \end{array}$$ (4.30)

Thus, the coefficients *b*_11_, *b*_12_, *b*_21_ and *b*_22_ (using the Eqs (4.24) and (4.30)) of the matrix *V* are

$$\begin{array}{c} \begin{array}{cc} & b_{11}=0,\;b_{12}=1,\;b_{21}=1,\;b_{22}=0. \end{array} \end{array}$$ (4.31)

The shape operator matrix coefficients of Eq (4.31) enable us to find the Gaussian-curvature and the mean-curvature of the biquadratic spacelike *q*-Bernstein-Bézier surface **s**(*u*, *v*) for *η* = −1 and they are,

$$\begin{array}{c} \begin{array}{cc} & K=1,\;H=0. \end{array} \end{array}$$ (4.32)

**Fig 4:**
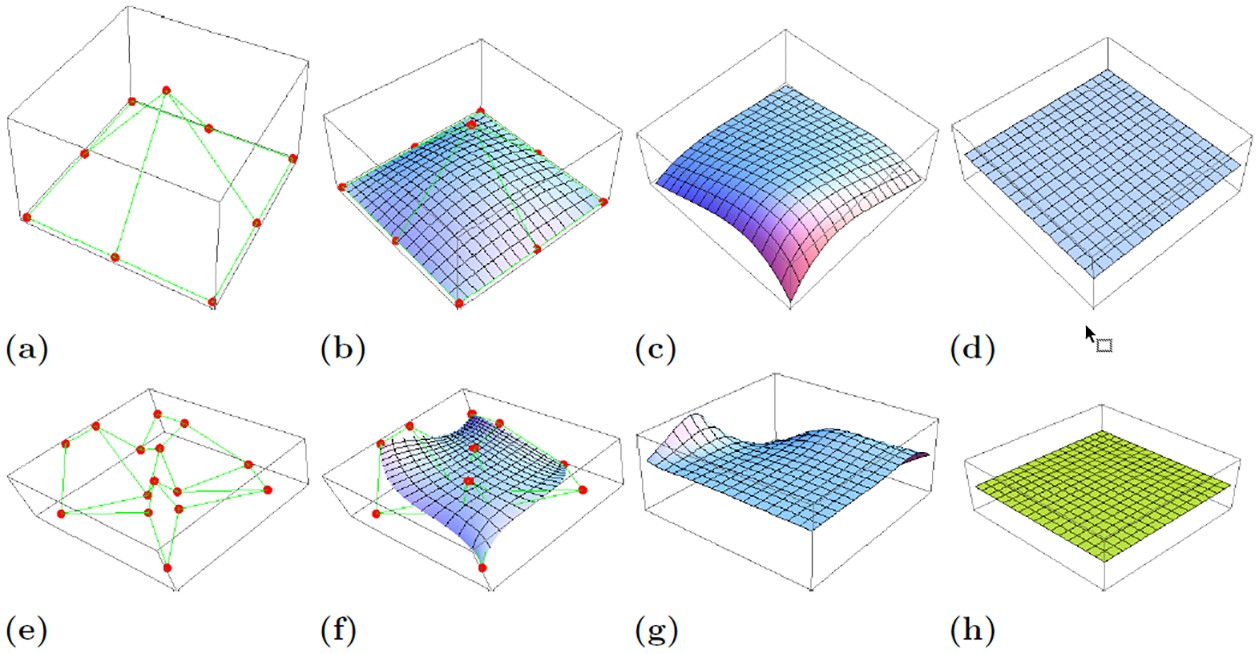
Figures (a)-(d) and (e)-(h) showcase the prescribed boundary, *q*-Bernstein-B’ezier surfaces for the spacelike biquadratic and bicubic cases respectively, along with their respective mean curvature at *q* = 0.2 and *q* = 1.

It is a minimal surface as in this case the mean curvature vanishes. For the prescribed boundary and the surface itself along with its mean curvature see Fig 4.

*Example* 4.4. **Shape-operator of Bicubic Spacelike** *q*-**Bernstein-Bézier Surface**

The control points ${♭}_{ȷ\ell }^{\left(1,0\right)}$ and ${♭}_{ȷ\ell }^{\left(0,1\right)}$ (Eqs (2.24) and (2.26)) for *ȷ*, *ℓ* = 0, 1 of the bicubic spacelike *q*-Bernstein-Bézier surface **s**(*u*, *v*) Eq (4.12) are,

$$\begin{array}{c} \begin{array}{cc} & {♭}_{00}^{\left(1,0\right)}=(-10,-7,0),\;{♭}_{01}^{\left(1,0\right)}=(-3,0,-6),\;{♭}_{10}^{\left(1,0\right)}=(-10,7,0),\\ & {♭}_{00}^{\left(0,1\right)}=(-7,-10,0),\;{♭}_{01}^{\left(0,1\right)}=(7,-10,0),\;{♭}_{10}^{\left(0,1\right)}=(0,-3,-6). \end{array} \end{array}$$ (4.33)

The coefficients (3.4) of the bicubic spacelike, *q*-Bernstein-Bézier surface (qbbs) in Minkowski space-$R_{1}^{3}$ are,

$$\begin{array}{c} E=149,\;F=144,\;G=149, \end{array}$$ (4.34)

and thus the corresponding metric of the bicubic spacelike *q*-Bernstein-Bézier surface **s**(*u*, *v*) is,

$$\begin{array}{c} ds^{2}=E\;du^{2}-2F\;du\;dv+G\;dv^{2}=149\;du^{2}+144du\;dv+149\;dv^{2}. \end{array}$$ (4.35)

The second-order partial derivatives (Eqs (2.35)–(2.37)) of bicubic spacelike case of *q*-Bernstein-Bézier surface **s**(*u*, *v*) are

$$\begin{array}{c} \begin{array}{cc} & s_{uu}\left(0,0\right)=(0,-59,0),\;s_{uv}\left(0,0\right)=(-44,-44,36),\;s_{vv}\left(0,0\right)=(-59,0,0). \end{array} \end{array}$$ (4.36)

Using the control points (4.33) in Eq (3.15), we can find the unit normal **N** to the bicubic spacelike *q*-Bernstein-Bézier surface (qbbs) as follows,

$$\begin{array}{c} N\left(0,0\right)=(0,0,1), \end{array}$$ (4.37)

and hence the norm of the unit normal **N** in this case,

$$\begin{array}{c} \varphi_{L}(N(0,0),N(0,0))=\eta =-1<0, \end{array}$$ (4.38)

indicates that it is a spacelike-vector. From the Eqs (4.36) and (4.37), the fundamental coefficients *e*, *f*, *g* of bicubic spacelike *q*-Bernstein-Bézier surface (qbbs) are

$$\begin{array}{c} \begin{array}{cc} & e=0,\;f=-36,\;g=0. \end{array} \end{array}$$ (4.39)

Substituting the values of the fundamental coefficients from Eq (4.34) in det (*ω*) (Eq (2.5)), we find that

$$\begin{array}{c} \text{det}(\omega )=1465>0. \end{array}$$ (4.40)

Now, the coefficients *b*_11_, *b*_12_, *b*_21_ and *b*_22_ (by virtue of the Eqs (4.34) and (4.40)) of the matrix *V* corresponding to the shape-operator of the bicubic spacelike *q*-Bernstein-Bézier surface (qbbs) are,

$$\begin{array}{c} \begin{array}{cc} & b_{11}=\frac{5184}{1465},\;b_{12}=-\frac{5364}{1465},\;b_{21}=-\frac{5364}{1465},\;b_{22}=\frac{5184}{1465}. \end{array} \end{array}$$ (4.41)

Gaussian-curvature and the mean-curvature of bicubic spacelike *q*-Bernstein-Bézier surface **s**(*u*, *v*) for shape operator matrix coefficients Eq (4.41) and for *η* = −1, are

$$\begin{array}{c} \begin{array}{cc} & K=\frac{1296}{1465},\;H=\frac{5184}{1465}. \end{array} \end{array}$$ (4.42)

The accompanying Fig 4 indicates the prescribed boundary for given control points, bicubic spacelike *q*-Bernstein-Bézier surface **s**(*u*, *v*) and its mean curvature for *q* = 0.2, 1.

## 5 Conclusion

In this paper, we present a family of Bézier surfaces called *q*-Bernstein-Bézier surfaces, in $R_{1}^{3}$-Minkowski space. We investigate the shape operators for the non-degenerate cases of these surfaces and provide illustrative examples of biquadratic and bicubic degenerate *q*-Bernstein-Bézier surfaces. The techniques used to find the shape operators of *q*-Bernstein-Bézier surfaces in Minkowski space are promising for further analysis in the field of differential geometry. The findings of this study can be useful in optimizing the shape of these surfaces to fit the requirements of a computational model for a surface, particularly in areas such as computer-aided geometric design and computer graphics.
